# Bioinformatics analysis and experimental validation of m6A and cuproptosis-related lncRNA NFE4 in clear cell renal cell carcinoma

**DOI:** 10.1007/s12672-024-01023-y

**Published:** 2024-05-26

**Authors:** Rui Feng, Haolin Li, Tong Meng, Mingtian Fei, Cheng Yang

**Affiliations:** 1 Department of Urology, the First Affiliated Hospital of Anhui Medical University, Anhui Medical University, Hefei, China; 2 Institute of Urology, Anhui Medical University, Hefei, Anhui China; 3 Anhui Province Key Laboratory of Urological and Andrological Diseases Research and Medical Transformation, Anhui Medical University, Hefei, Anhui China

**Keywords:** Clear cell renal cell carcinoma, Cuproptosis, M6A, Long non-coding RNA, Prognostic mode

## Abstract

**Purpose:**

This study aimed to construct an m6A and cuproptosis-related long non-coding RNAs (lncRNAs) signature to accurately predict the prognosis of kidney clear cell carcinoma (KIRC) patients using the information acquired from The Cancer Genome Atlas (TCGA) database.

**Methods:**

First, the co-expression analysis was performed to identify lncRNAs linked with N6-methyladenosine (m6A) and cuproptosis in ccRCC. Then, a model encompassing four candidate lncRNAs was constructed via univariate, least absolute shrinkage together with selection operator (LASSO), and multivariate regression analyses. Furthermore, Kaplan–Meier, principal component, functional enrichment annotation, and nomogram analyses were performed to develop a risk model that could effectively assess medical outcomes for ccRCC cases. Moreover, the cellular function of NFE4 in Caki-1/OS-RC-2 cultures was elucidated through CCK-8/EdU assessments and Transwell experiments. Dataset outcomes indicated that NFE4 can have possible implications in m6A and cuproptosis, and may promote ccRCC progression.

**Results:**

We constructed a panel of m6A and cuproptosis-related lncRNAs to construct a prognostic prediction model. The Kaplan–Meier and ROC curves showed that the feature had acceptable predictive validity in the TCGA training, test, and complete groups. Furthermore, the m6A and cuproptosis-related lncRNA model indicated higher diagnostic efficiency than other clinical features. Moreover, the NFE4 function analysis indicated a gene associated with m6A and cuproptosis-related lncRNAs in ccRCC. It was also revealed that the proliferation and migration of Caki-1 /OS-RC-2 cells were inhibited in the NFE4 knockdown group.

**Conclusion:**

Overall, this study indicated that NFE4 and our constructed risk signature could predict outcomes and have potential clinical value.

**Supplementary Information:**

The online version contains supplementary material available at 10.1007/s12672-024-01023-y.

## Introduction

Renal cell carcinoma (RCC) ranks third most prevalent urinary system malignancy [1], with clear cell renal cell carcinoma (ccRCC) as its predominant subtype [2]. Despite the advances in therapeutic interventions for RCC, radical nephrectomy continues to be the primary treatment [3]. In comparison to other RCC subtypes, ccRCC exhibits radio/chemoresistance and has a higher risk of recurrence and metastasis. Therefore, elucidating the underlying molecular mechanisms of ccRCC pathogenesis would allow the development of efficient therapies and the identification of a robust prognostic signature for ccRCC survival outcome prediction.

Nucleotide methylation is considered to be a cardinal epigenetic mechanism governing cellular development and differentiation [4]. Of these, N6-methyladenosine (m6A) modification is an epigenetic change in mRNAs and noncoding RNAs [5], that is essentially involved in different biological processes such as RNA splicing, export, and translation. Furthermore, m6A has also been associated with the development and progression of cancers [6]. Such versatile regulation of m6A modification is mediated by regulators, including methyltransferase complexes (“writers”), signal transducers (“readers”), and demethylases (“erasers”) [7]. For instance, the m6A methyltransferase METTL3 promoted the growth of prostate cancer cells via the Hedgehog pathway [8], while ALKBH5, an RNA demethylase, inhibited the progression of pancreatic cancer by regulating the post-translational PER1 activation [9]. Studies have also revealed the critical roles played by m6A-regulated genes in ccRCC pathogenesis. Zhuang et al. [10] showed the involvement of FTO in the suppression of ccRCC via PGC-1a. Moreover, according to Gao et al. [11], DMDRMR-regulated CDK4 plays an important role in ccRCC progression through the m6A reader IGF2BP3.

Cuproptosis is a unique biological process triggered by copper (Cu) [12], a vital mineral nutrient associated with mitochondrial respiration, antioxidation, and iron uptake [13]. It has been previously indicated that protein lipidation mediates copper-induced cell death, and excess copper can promote the aggregation of lipid proteins, disrupt the stability of Fe-S cluster proteins, and induce protein toxic stress, ultimately causing cell death [14]. A study on cuproptosis revealed that solute carrier family 31 member 1 (SLC31A1) and copper transporter P-type ATPases (ATP7A and ATP7B) are key molecules required to regulate and maintain mammalian copper homeostasis [15, 16]. Much literature suggests that the levels of Cu are elevated in various malignant cancer tumors than in healthy tissue counterparts, such as in cancers of cervical, thyroid, ovarian, lung, pancreatic, prostate, gastric, oral, and bladder [17–26]. Cu-binding compounds can serve as potential anticancer agents [27]. Furthermore, various classes of Cu ionophores, including flavones, bis(thiosemicarbazone) ligands, 8-hydroxyquinolines, and dithiocarbamate have been identified for their abilities to promote cuproptosis and mediate anticancerous functions [27–31]. Consequently, novel Cu-binding molecules that selectively affect tumor cells are being actively researched. Nevertheless, the association between cuproptosis and ccRCC remains unclear and warrants further investigation.

Long-noncoding RNAs (lncRNAs) became a research hotspot because of their association with different eukaryotic biological processes [32]. These have also been observed to induce carcinogenic properties, including proliferation, differentiation, apoptosis, drug resistance, and metastasis in cells [33, 34]. However, whether lncRNAs are involved in m6A and cuproptosis remains unknown and their identification may speed up diagnoses and prognoses for ccRCC patients. Recent studies have analyzed m6A and cuproposis-associated lncRNAs in renal cancer [35, 36]; however, only a few studies have explored their roles in ccRCC.

Therefore, this investigation performed bioinformatics and statistical analyses to develop a prognostic signature framed on lncRNAs specific to m6A and cuproptosis using ccRCC case data from The Cancer Genome Atlas (TCGA) database. Furthermore, this study aimed to apply this signature for accurate prediction of prognostic outcomes in ccRCC. The developed signature comprised four m6A- and cuproptosis-related lncRNAs, which demonstrated high predictive ability. Moreover, the overall survival (OS) of ccRCC patients was also quantitatively predicted by constructing a nomogram. In addition, further functional enrichment and gene mutation analyses were performed to support these findings. The functions of NFE4 were investigated and one of four identified functions associated with ccRCC was selected for further study.

## Materials and methods

### Data collation

The expression, clinical, and mutation data of ccRCC patients were downloaded from TCGA (https://gdc.cancer.gov/) from inception till April 30, 2023. To minimize errors in data assessment and reduce statistical bias, this investigation eliminated all ccRCC patients with missing OS or clinical data. The acquired clinical data included age, sex, degree of tumor differentiation, TNM stage, and survival time/status.

### Screening of m6A and cuproptosis-related lncRNAs

The expression profile for lncRNA, m6A, and cuproptosis genes were imported from TCGA. Based on previous studies, 29 genes associated with m6A were identified, including ZC3H13, YTHDF3, YTHDF2, YTHDF1, YTHDC2, YTHDC1, WTAP, VIRMA, RBMX, RBM15B, RBM15, NSUN2, NKAP, MTCH2, METTL3, METTL16, METTL14, LRPPRC, IGF2BP3, IGF2BP2, IGF2BP1, HNRNPC, HNRNPA2B1, FTO, FMR1, EIF3A, CBLL1, ALKBH5, and ALKBH3. Furthermore, 19 cuproptosis genes were also identified, these included NFE2L2, NLRP3, ATP7B, ATP7A, SLC31A1, FDX1, LIAS, LIPT1, LIPT2, DLD, DLAT, PDHA1, PDHB, MTF1, GLS, CDKN2A, DBT, GCSH, and DLST. Moreover, 6366 m6A-associated and 1063 cuproptosis- related lncRNAs were screened via Pearson’s correlation assessment and then intersected to detect 1017 target genes based on | Pearson R|> 0.4 and p < 0.05.

### Grouping and risk model construction

We randomly divided the study population into training and testing sets and employed a training set for developing a prognostic model of lncRNAs linked with m6A and cuproptosis. Statistical assessment revealed no major differences in clinical characteristics between the two cohorts (p > 0.05, Table S1). Initially, lncRNAs were screened for Cox univariate analysis, and then the R “glmnet” [37] package was employed for least absolute shrinkage and selection operator (LASSO) regression evaluations. Subsequently, multivariate Cox regression was performed to obtain four candidate lncRNAs and a prognostic risk model was constructed through: Risk score = 0.10952189 × NFE4 + 0.341981448 × LINC02154 +  (− 0.490148161) × AL161782.1 +  (− 0.782907284) × AL355835.1. Based on the median risk scores the cases were categorized into low-risk and high-risk cohorts.

### Cox regression assessment for m6A- and cuproptosis-related lncRNA models

To determine if other clinical features such as age, sex, grade, stage, TNM stage, and risk score serve as independent prognostic factors within the risk model, univariate and multivariate Cox regression assessments were performed after the data was collected.

### Construction of survival curves and principal component assessment (PCA)

To evaluate variations within OS and progression-free survival (PFS) among both risk cohorts, the Kaplan–Meier (K-M) test was performed using the “survival” [38] and “survminer” [39] package in R. Then, PCA was carried out to effectively reduce the dimensionality for the lncRNA dataset linked with m6A and cuproptosis. Lastly, the compressed data were analyzed and visualized through “limma” [40] and “scatterplot3d” [41] packages, respectively.

### Functional and model analyses in immunotherapy

We identified the functions of differentially expressed genes by performing gene ontology (GO) assessment through “clusterProfiler.” [42] The threshold for significant functional enrichment was set as p < 0.05. After downloading tumor mutation data, the R “maftools” [43] package was used to evaluate the tumor mutational burden (TMB) within two cohorts. The cutoff value used to distinguish between high and low TMB is the median, which is 1.552631579. Moreover, a tumor immune dysfunction and exclusion (TIDE) assay was performed to predict the sensitivity of immunotherapy in the two cohorts.

### Building a nomogram

We generated a predictive nomogram through predictors such as age, sex, risk score, grade, and TNM stage for predicting 1, 3, and 5-year OS. Additionally, the Hosmer–Lemeshow test was performed and a calibration curve was developed to depict agreement for observed outcomes with model predictions.

### Tissue samples analysis

From May 2019 to March 2021, 45 ccRCC and neighboring (≥ 2 cm away) healthy renal tissue samples were surgically collected from the Department of Urology, The First Affiliated Hospital of Anhui Medical University (Hefei, China). Two pathologists histologically diagnosed the type of ccRCC, independently. Written informed consent was acquired for all patients and the study was approved by the Ethics Committee of Human Research for First Affiliated Hospital of Anhui Medical University (No. PJ2019-14-22).

### Chemicals and reagents

The following is a list of the supplies and antibodies used in this experiment: we purchased copper ionophore Elesclomol and copper chloride from MedChemExpress from NJ, USA, while Aladdin from Shanghai, China. The primary anti-ATP7B (AF0410), anti-SLC31A1 (DF13356), and anti-beta Actin (AF7018) antibodies were acquired from the Affinity Biosciences. HRP-conjugated goat anti-rabbit (SA00001–2) secondary antibody was provided by ProteinTech.

### RNA extraction and qRT-PCR

Total RNA from the tissue was extracted through TRIzol^®^ Reagent BD (Invitrogen^™^, USA), and its concentration and purity were determined through a NanoDrop 2000^®^ spectrophotometer (NanoDrop Technologies^™^, USA). Acquired RNA (2 μg) was reverse transcribed using a PrimeScript^®^ RT reagent kit (Takara^™^, Japan), per the kit’s instruction. Consequently, cDNA was subjected to qPCR through SYBR Green Mix (Takara^™^, Japan) across the ABI7500^®^ system (Thermo^™^, USA). The relative expression of NFE4 was normalized by the 2^−ΔΔCT^ method and *GAPDH* was used as a normalization gene. Primers were chemically synthesized by Sangon Biotech^®^ (Sangon^™^, China). Primer sequences were: NFE4, (forward) 5′-CTTGTGCCAGCAGTGTGAGTC-3′ and (reverse) 5′-CGTGTCTCCCAGTCAGAGTAGG-3′; GAPDH, (forward) 5′-TTGCCCTCAACGACCACTTT-3′ and (reverse) 5′-TGGTCCAGGGGTCTTACTCC-3′.

### Cell culture and transfection

The 786-O, A498, OS-RC-2, Caki-1, ACHN, and HK-2 cell cultures were procured from the Chinese Academy of Medical Sciences (Shanghai, China). All except ACHN cells were grown in high-glucose Dulbecco’s minimum essential medium (DMEM; Gibco) augmented with 10% fetal bovine serum (FBD) and 1% penicillin–streptomycin solution, while ACHN culture was grown within MEM medium (Gibco). All cellular cultures were maintained at 37 °C and 5% CO_2_ in fresh medium (replaced daily) and passaged until 70% confluency. Two interference sequences that specifically target NFE4 (siRNA# 1: 5′-GACAATTCCTGTTTACGGAAGACTA-3′ and siRNA# 2: 5′-CAATTCCTGTTTACGGAAGACTATA-3′) and negative control (si-NC: 5′-TTCTCCGAACGTGTCACGT-3′) siRNA (Cat #: P202101170036) were developed through RiboBio^™^ (China). Furthermore, OS-RC-2 and Caki-1 cells were transfected with 60 nM of siNFE4 or si-NC via Lipofectamine 2000 (Invitrogen^™^), and a final cell suspension volume of 100 nM was cultured.

### Cell drug treatment

When the renal cancer cell lines (OS-RC-2 and Caki-1) were adherent and morphologically diffused, they were treated with 2 mM copper chloride or 20 nM Elesclomol. After 24 h of treatment, cells were collected, and RNA was isolated.

### Cell proliferation assessment

Cells were cultured in a 96-well plate (1000 cells/well) at 37 °C. For each cohort, five replicate and blank control wells (containing 200 μL FBS-free medium) were prepared. The spent medium was daily replaced with fresh medium. The cells were then placed into incubation with 10 μL of Cell Counting Kit-8 (CCK8; Dojindo Molecular Technology) reagent at 37 °C in the dark. Absorbance (OD450) was assessed daily and determined for 4 days via a microplate reader. Cell proliferation was analyzed through cell count per day and displayed as a line graph.

For the EdU assessment, cells were seeded in 24-well plates and subsequently exposed to EdU (Beyotime Biotechnology, China). Images were acquired using fluorescence microscopy (Olympus^™^, Japan) and processed via Image J. Furthermore, fluorescent cell quantities and the degree of proliferating cells were determined.

### Western blotting assay

Caki-1 and OS-RC-2 Cells were dissolved in RIPA buffer (radioimmunoprecipitation assay solution) augmented with protease and phosphatase inhibitors, as well as PMSF. The denatured total protein samples were then subjected to PAGE (polyacrylamide-sodium dodecyl sulfate gels) and transferred onto the nitrocellulose membrane. The membranes’ non-specific binding sites were inhibited with 5% (w/v) defatted milk. Subsequently, the membranes were treated with primary antibodies at 4 °C for 12 h, washed thrice, and then tagged with horseradish peroxidase coupled secondary antibodies for two hours. Finally, using an EZ-ECL kit was used to view the membranes.

### Transwell migrative and invasive property assessments

Cell migration and invasion properties were studied in a 24-well Transwell chamber with 8 μm pores (Costar^™^, Germany). Briefly, Caki-1 and OS-RC-2 cells were transfected with NFE4-specific siRNAs before the experiment and incubated in a serum-free medium for 8 h. For migration assessment, 600 μL of 10% FBS-supplemented medium was introduced within the lower chamber (i.e. bottom of 24-well plate), while 200 μL cell suspension was introduced within the upper chamber. Plates were incubated for 1 day. For invasion analysis, the upper wells of a 24-well plate were coated in Matrigel (diluted in serum-free medium 1:8; BD Biosciences) at 37 °C for 8 h. Consequently, 600 μL 10% FBS-supplemented medium was introduced within lower chambers. The Transwell chamber was placed with tweezers, while 200 μL cell suspension was introduced within the upper chambers. After 24 h of incubation, the medium was aspirated, and cells remaining in the upper Matrigel surface and upper chamber were gently wiped through cotton swabbing, fixed in 4% paraformaldehyde for 15 min, and stained with 0.05% crystal violet for 5–10 min.

### Statistical analysis

All statistical analyses were performed using R software (version 4.2.0), except for the statistical analysis of qPCR results, which were analyzed by the one-way analysis of variance (ANOVA) using GraphPad Prism (version 8.2.1). The intergroup differences were analyzed via the Wilcoxon rank sum test. Furthermore, univariate and multivariate Cox regression analyses were carried out to obtain independent predictors for RCC. *p* < *0.05* was set as the statistically significant value (*p < 0.05; **p < 0.01; ***p < 0.001).

## Results

### Identification of lncRNA genes associated with M6A and cuproptosis in ccRCC

**Figure. 1:**
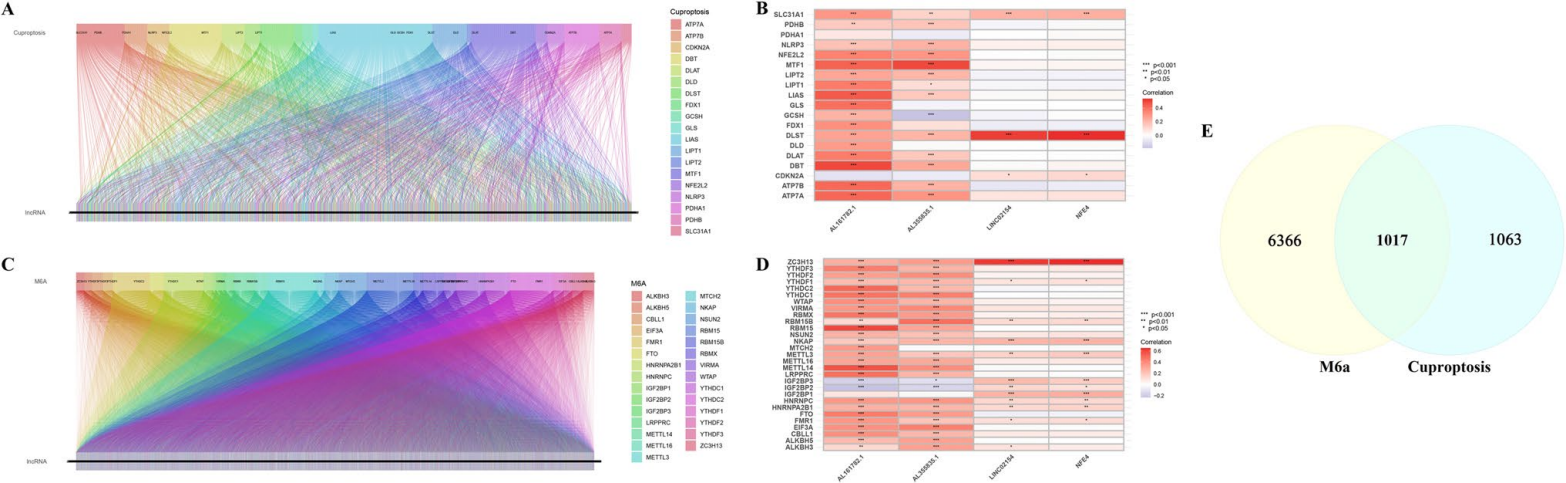
Identification of m6A-related lncRNAs and cuproptosis-related lncRNAs in RCC patients. **A** Sankey relational diagram for 19 cuproptosis genes and cuproptosis-related lncRNAs. **B** Heatmap of the correlations between 19 cuproptosis genes and the 4 cuproptosis-related lncRNAs. **C** Sankey relational diagram for 29 m6A genes and m6A-related lncRNAs. **D** Heatmap of the correlations between 29 m6A genes and the 4 prognostic m6A-related lncRNAs. **E** Relationship and number of m6A-related lncRNAs and cuproptosis-related lncRNAs.

We performed a Person’s correlation analysis of 29 m6A-linked and 19 cuproptosis-linked genes identified from TCGA. Then 6366 and 1063 m6A and cuproptosis-associated lncRNAs were screened, respectively, and depicted as Sankey plots (Fig. 1A and Fig. 1C). Furthermore, the developed heatmaps revealed the correlation of lncRNAs with m6A (Fig. 1B) and cuproptosis (Fig. 1D). The Venn plot shown in Fig. 1E demonstrates the intersection of two sets and highlights 1017 target lncRNAs.

### Construction of risk model for ccRCC

**Figure. 2:**
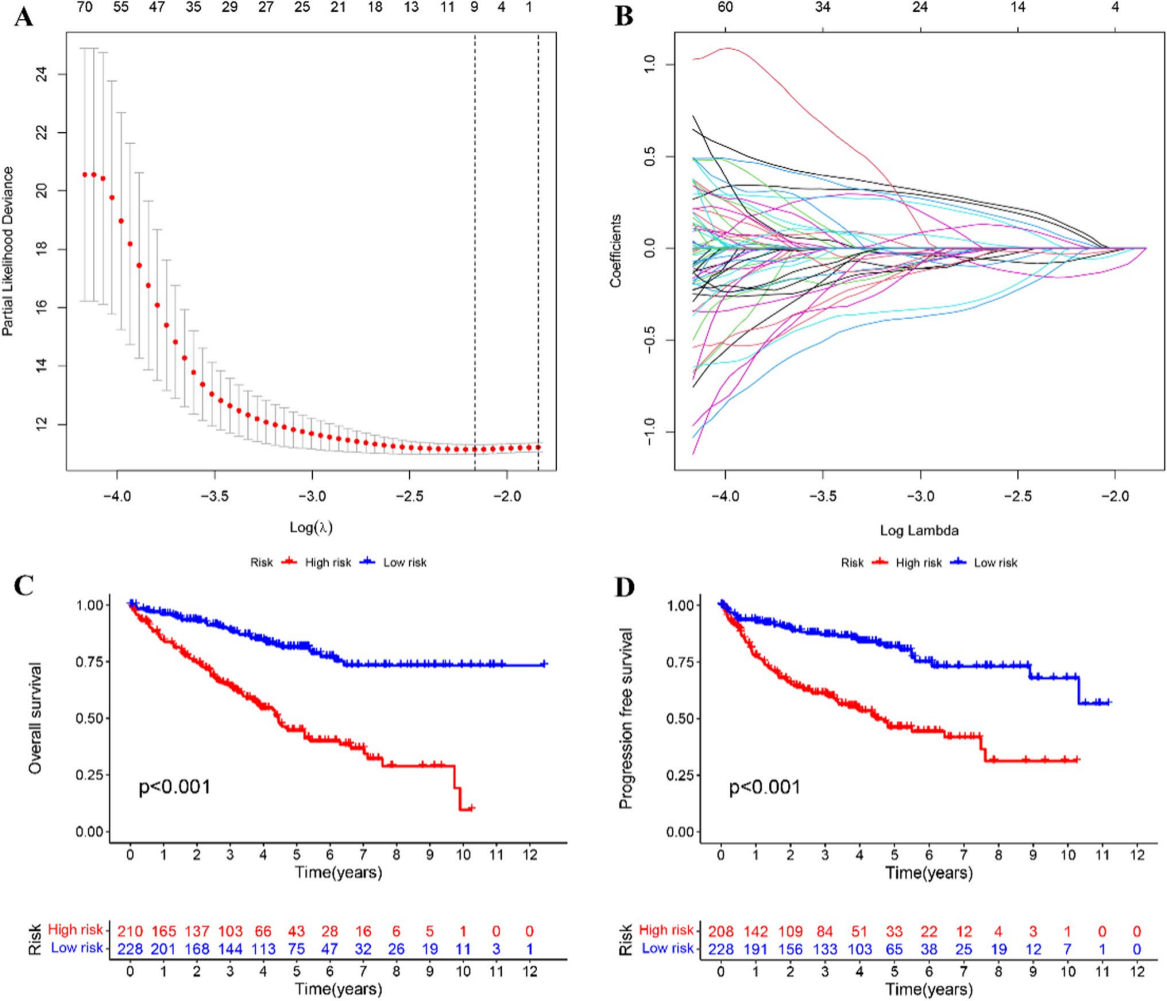
Risk model for RCC patients based on cuproptosis and m6A-related lncRNAs. **A** The tuning parameters (log λ) of OS-related proteins were selected to cross-verify the error curve. According to the minimal criterion and λ-se criterion, perpendicular imaginary lines were drawn at the optimal value. **B** The LASSO coefficient profile of 4 OS-related lncRNAs and perpendicular imaginary lines were drawn at the value chosen by 10-fold cross-validation. **C** Kaplan-Meier survival curves of the OS of patients in the high- and low-risk groups. **D** Kaplan-Meier survival curves of PFS of patients in the low- and high-risk groups for the entire set.

We first subjected 1017 lncRNAs to univariate regression followed by LASSO regression assessment (Fig. 2A and B), and lastly, the multivariate Cox regression analysis. The results identified four OS-linked lncRNAs, which were used to generate a risk model. Moreover, the prognostic risk of ccRCC was determined.

**Figure. 3:**
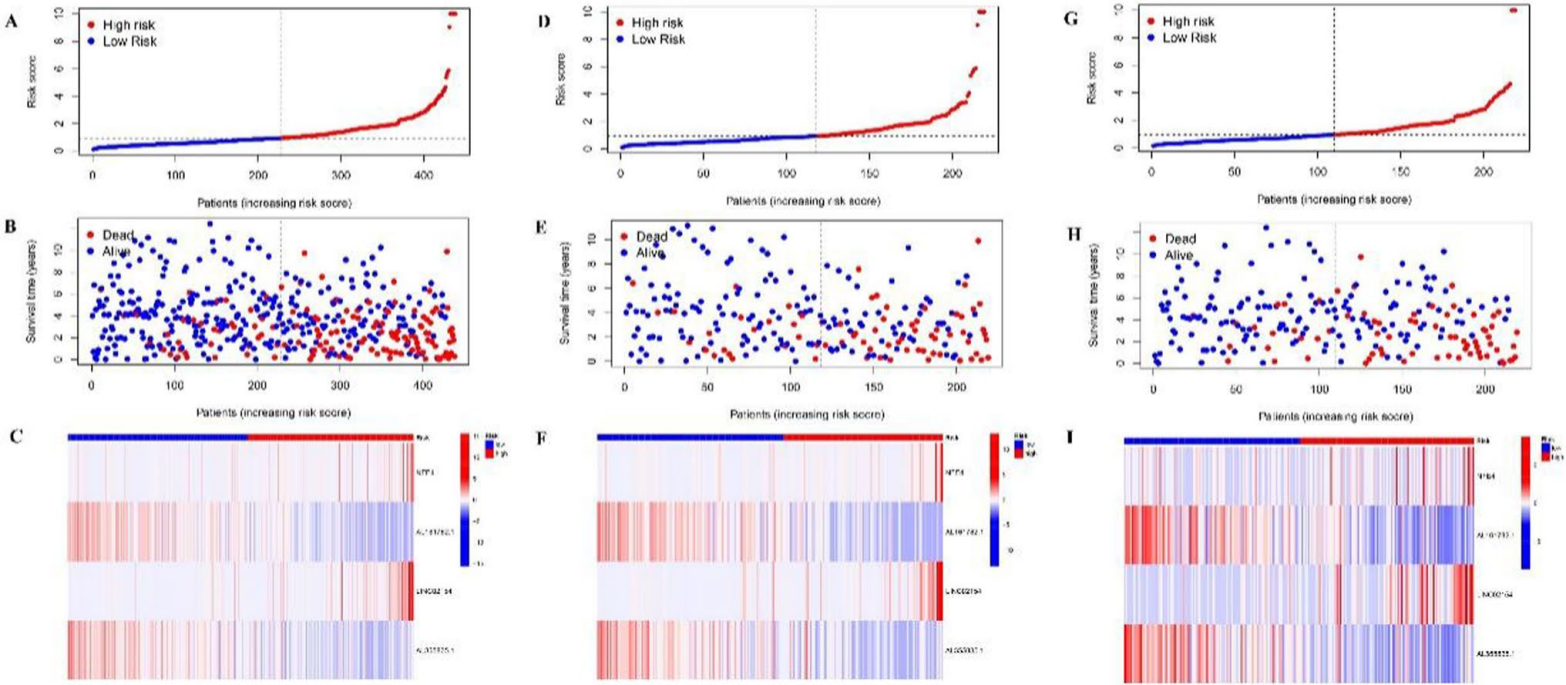
Prognostic value of the risk model of the 4 cuproptosis and m6A-related lncRNAs in the TCGA testing and entire sets. **A** Distribution of cuproptosis-related lncRNA model-based risk scores for the testing set. **B** Patterns of the survival time and survival status between the high- and low-risk groups for the testing set. **C** Clustering analysis heatmap shows the display levels of the 4 cuproptosis-related lncRNAs for each patient in the testing set. **D** Distribution of the m6A-related lncRNA model-based risk score for the testing set. **E** Patterns of the survival time and survival status between the high- and low-risk groups for the testing set. **F** Clustering analysis heatmap shows the display levels of the 4 m6A-related lncRNAs for each patient in the testing set. **G** Distribution of cuproptosis and m6A-related lncRNA model-based risk scores for the testing set. **H** Patterns of the survival time and survival status between the high- and low-risk groups for the testing set. **I** Clustering analysis heatmap shows the display levels of the 4 cuproptosis- and m6A-related lncRNAs for each patient in the testing set.

Based on the median risk values, the cases were grouped into high and low-risk cohorts. Figure 2C shows the effect of the model on the OS of these two cohorts, while Fig. 2D illustrates their PFS. Survival assessment revealed improved OS and PFS in the low-risk cohort than in the high-risk cohort (*p* < *0.001*). Risk levels spread across both cohorts (Fig. 3A), and their survival status and time are shown in Fig. 3B. Figure 3C indicates a heatmap of four lncRNA expressions per patient from TCGA. To ensure the robustness of the model, randomization was applied to categorize patients into the experimental and control sets, and then the results were calculated through a unified formula. The statistical assessment of clinical data revealed no marked variation across high- and low-risk cohorts (Table S1). Figure 3D, E, F and G, H, I display the risk level distribution among experimental and control sets, their survival status and time, as well as the lncRNA expression heatmap, respectively.

### Assessment of clinical features in the risk model

**Figure. 4:**
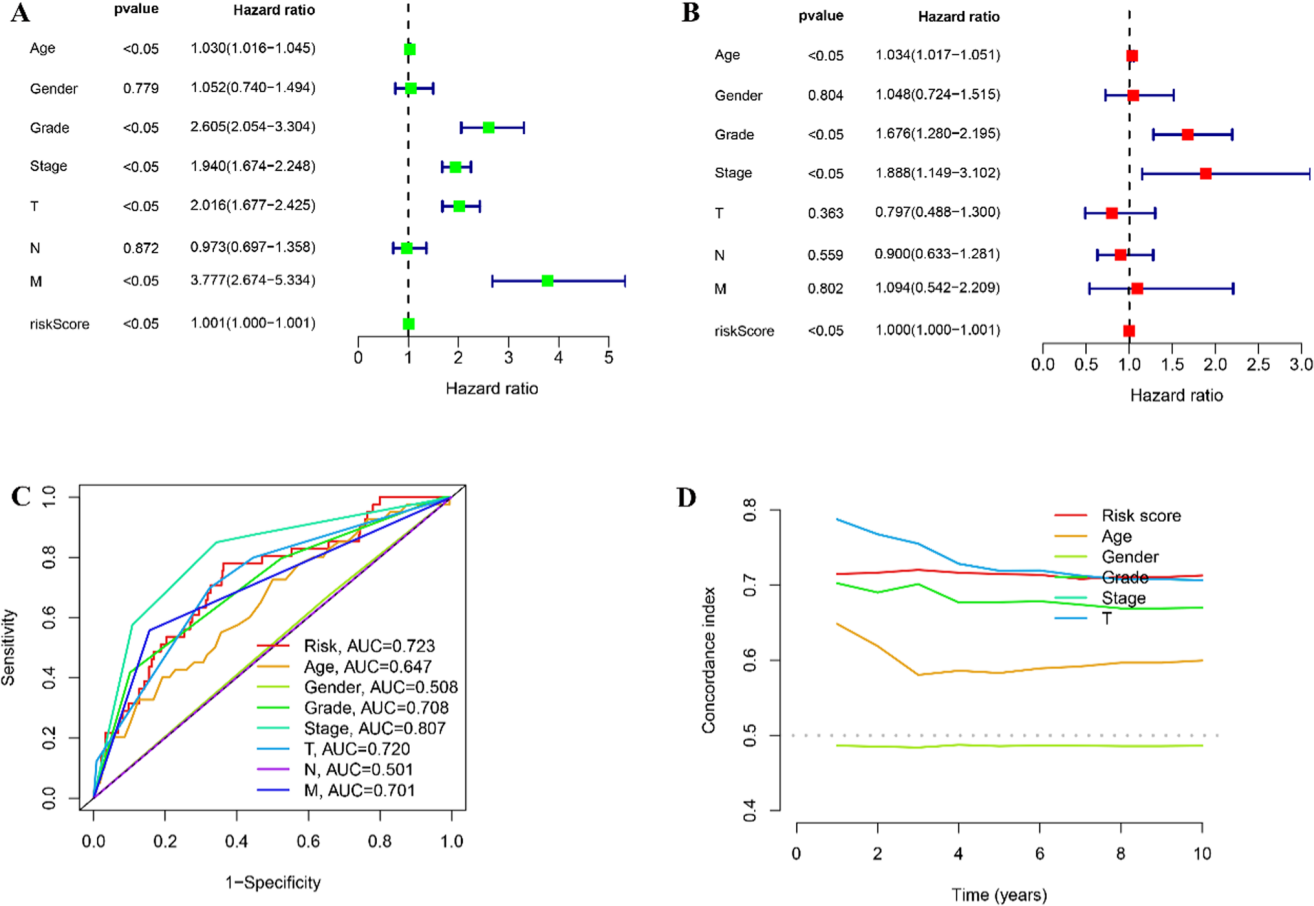
Assessment of the prognostic risk model of cuproptosis and m6A-related lncRNAs and clinical features in RCC in the entire TCGA dataset. **A** Univariate and multivariate analyses of the clinical characteristics and risk score with OS. **B** Univariate and multivariate analyses of the clinical characteristics and risk score with OS. **C** ROC curves of the clinical characteristics and risk score. **D** Concordance indexes of the risk score and clinical characteristics.

The risk score, age, sex, TNM stages, and grade were included in the univariate and multivariate assessments. Univariate assessment revealed a correlation with all the factors except for sex (p = 0.779) and N stage (p = 0.872) (p < 0.05, HR: 1.001, 95% CI 1.000–1.001) (Fig. 4A). Whereas, multivariate assessment indicated that the risk score was related only to age, grade, and stage (HR: 1.001, 95% CI 1.000–1.001; Fig. 4B) though not with sex, T stage, N stage, and M stage. Furthermore, the area under the receiver operator characteristic (ROC) curve (AUC) (Fig. 4C) and compliance index (Fig. 4D) were evaluated for assessing risk score. Moreover, the model’s specificity and sensitivity in predicting ccRCC prognosis were assessed. The risk score had an AUC curve value of 0.723, second only to grade staging. Although the compliance index was initially lower than the T stage, it gradually approached the T stage over time. Thus, the four lncRNAs were relatively reliable markers for developing a ccRCC prognostic risk model.

**Figure. 5:**
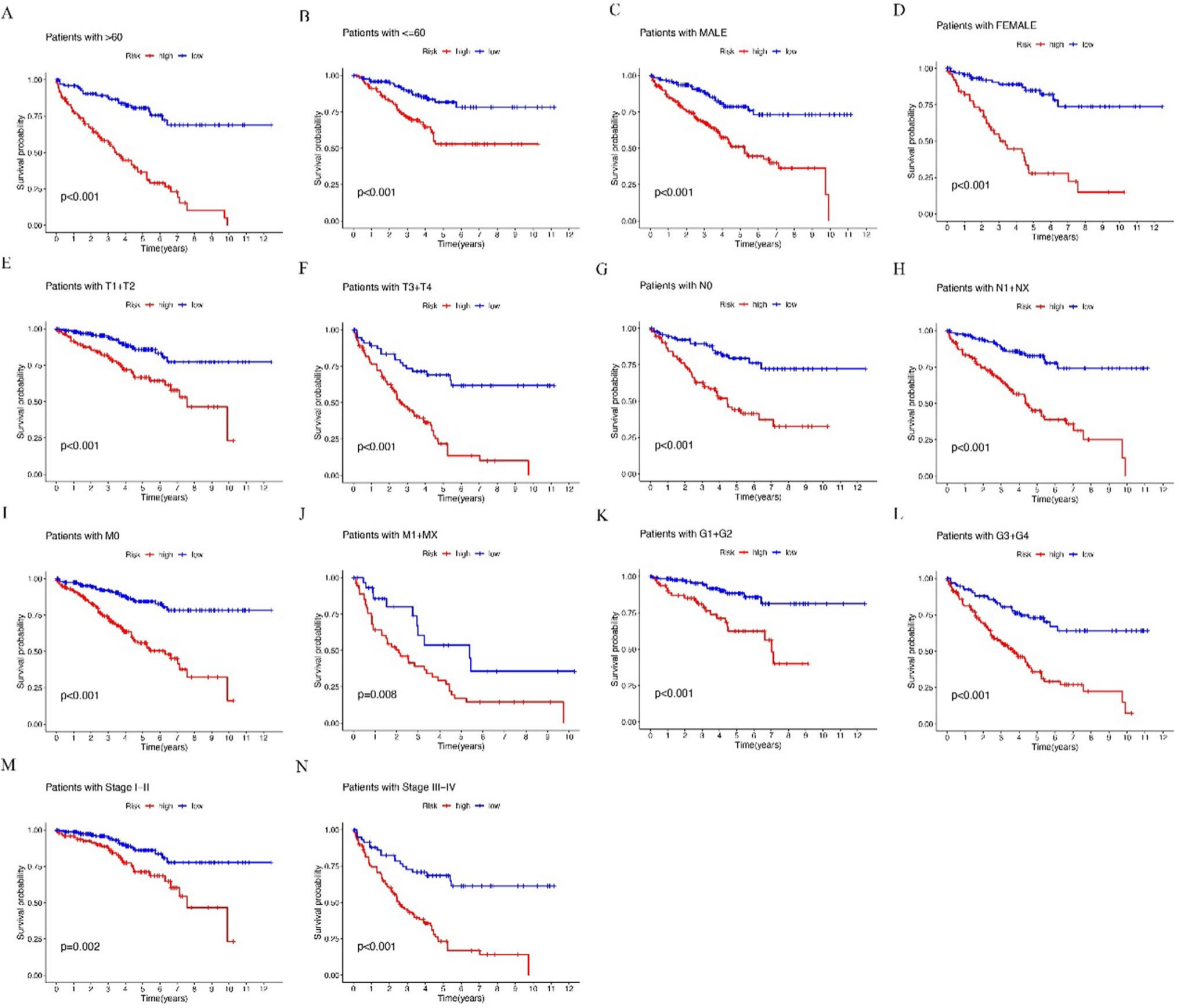
Kaplan‒Meier curves of OS differences stratified by sex, age, tumor grade, or TNM stage between the high and low-risk groups in the TCGA entire set.

This investigation revealed that there were differences in OS between the high- and low-risk cohorts, and the OS was more improved in the low-risk cohort than the high-risk cohort (Fig. 5A–N).

### Nomogram construction and evaluation

**Figure. 6:**
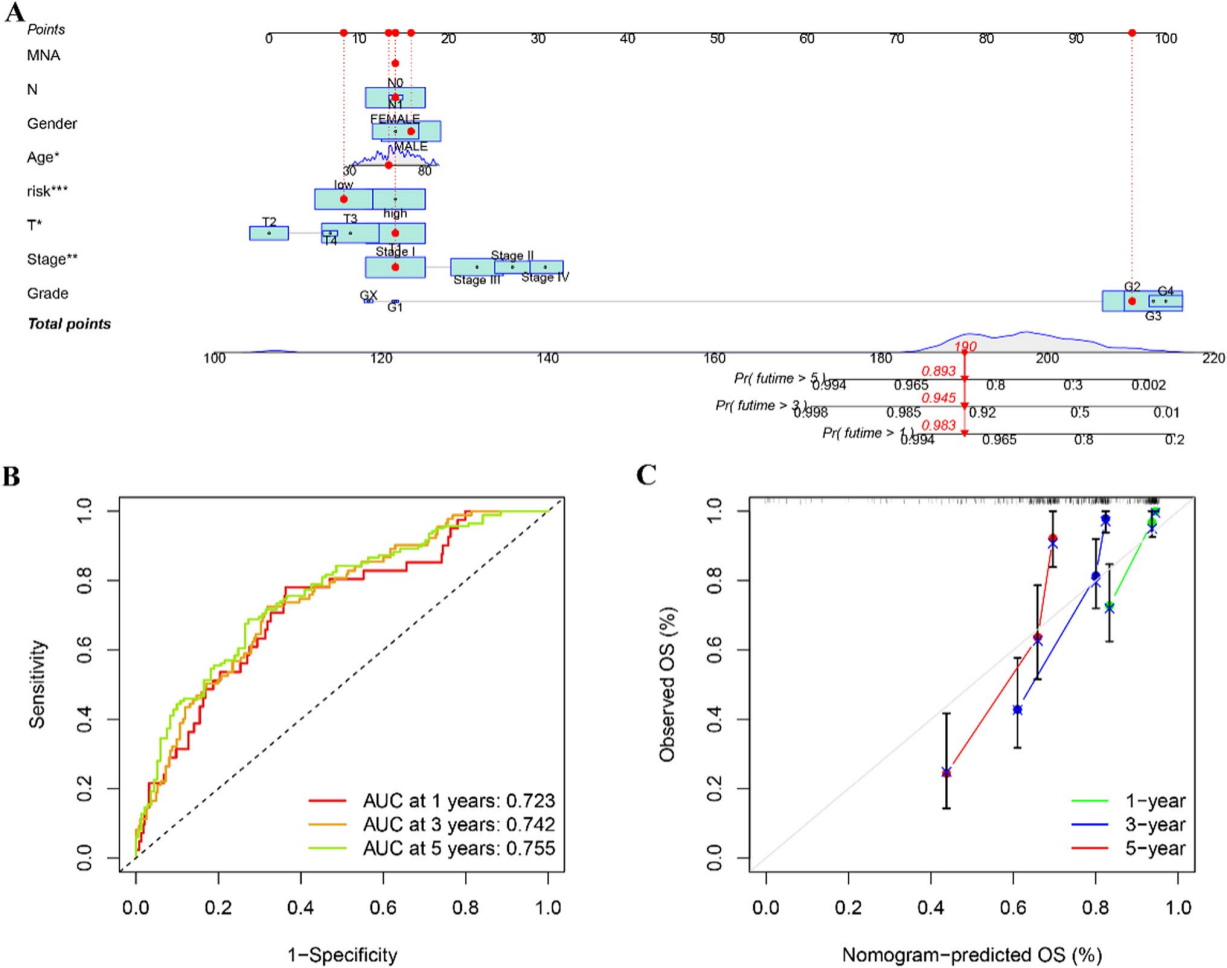
Build and evaluate a nomogram based on clinicopathological factors. **A** Prognostic nomograms were constructed based on our risk model and other prognosis-related clinical elements. **B** ROC curves were drawn to evaluate the predictive ability of the nomogram for 1, 3, and 5-year overall survival in KIRC patients. **C** Calibration curves showed good agreement between predicted and observed survival rates.

To predict the OS, the nomogram plots were constructed using the risk scores and seven clinically characteristic pathologies. Risk scoring capacity for assessing prognostic OS (Fig. 6A) was evaluated by comparing it with other clinicopathological factors. The prediction results of AUC were consistent (Fig. 6B), while predictions at 1, 3, and 5 years were still relatively near the ideal curve, suggesting its satisfactory calibration accuracy (Fig. 6C).

### Grouping capability of the PCA model

**Figure. 7:**
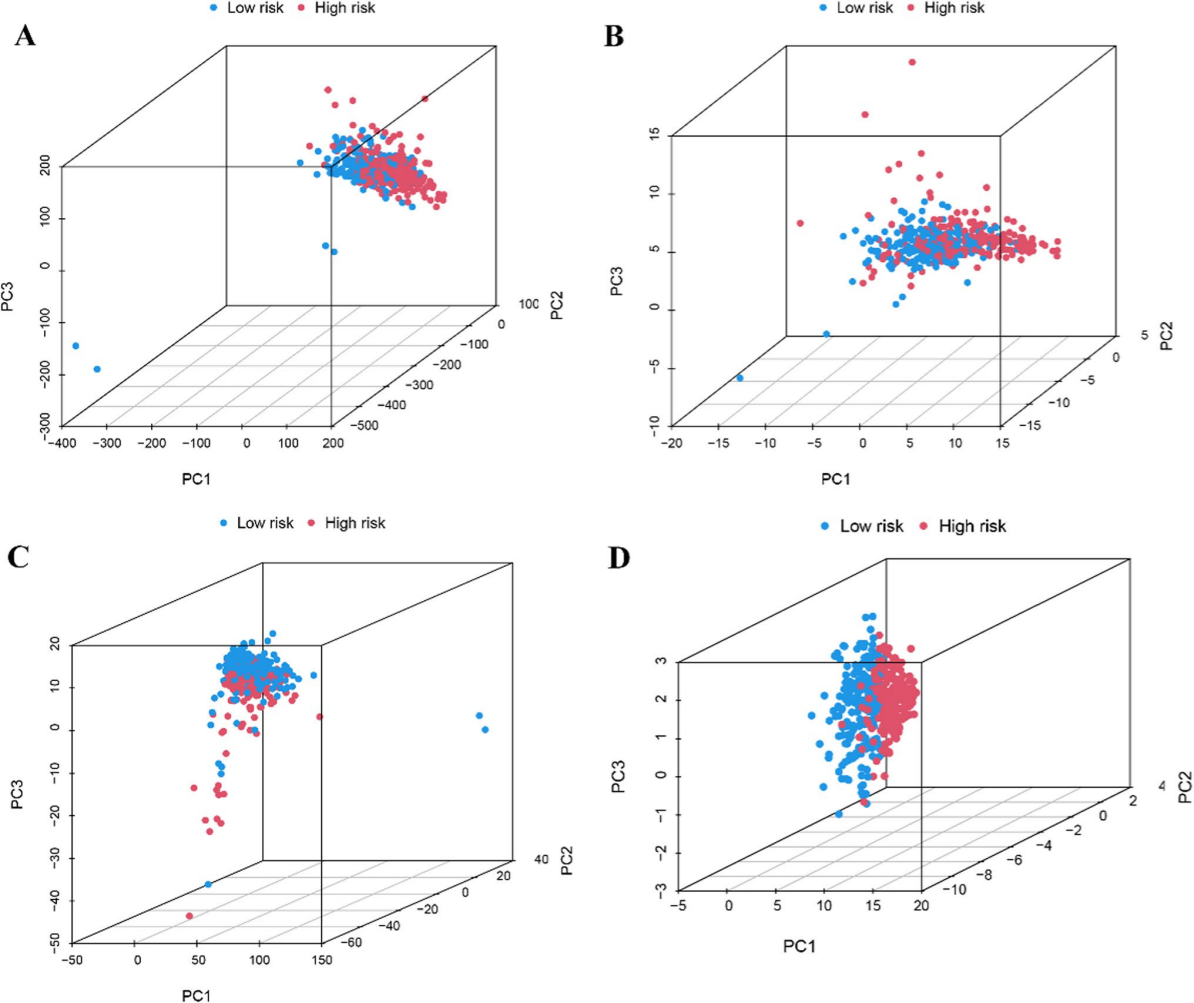
Principal component analysis between the high and low-risk groups based on. **A** entire gene expression profiles, **B** 29 m6A genes plus 19 cuproptosis genes, **C** m6A and cuproptosis -related lncRNAs, and **D** risk model based on the representation profiles of the 4 cuproptosis and m6A-related lncRNAs in the TCGA entire set.

PCA provides intuitive observation of similarities among individual samples. Therefore, PCA was performed to test differences among the two risk cohorts, based upon the following datasets: whole gene expression profiles from TCGA, 29 m6A genes + 19 cuproptosis genes, four candidate lncRNAs, while risk model (Fig. 7A, B, C, D). Figure 7A, B, C show the well-separated distribution for high- and low-risk cohorts. Figure 7D indicates the difference in their distribution and highlights the strength of this prognostic model.

### Immune microenvironment and sensitivity of ccRCC to immunotherapy

**Figure. 8:**
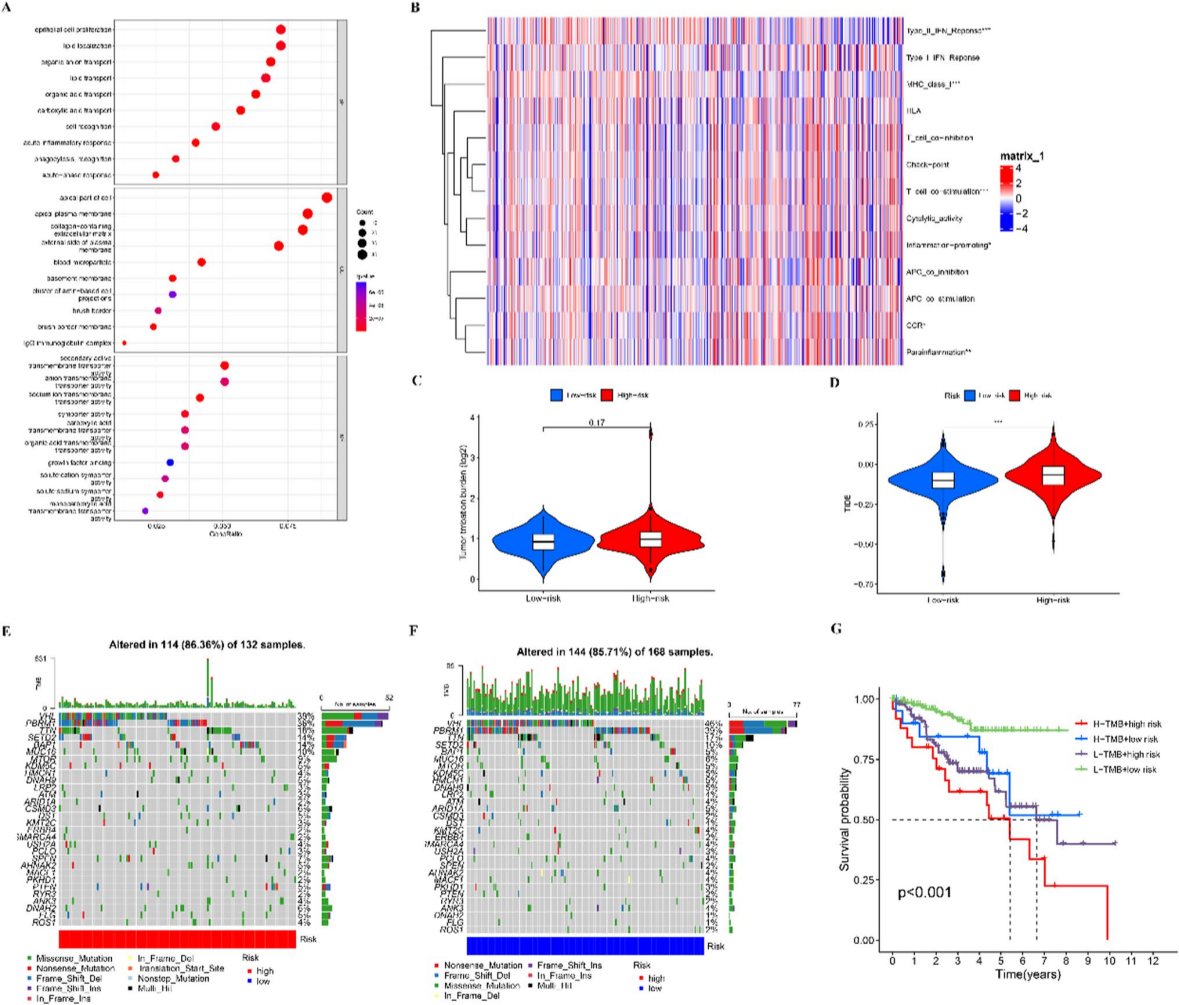
Gene enrichment and tumor mutation analysis. **A** GO enrichment analysis for DEGs between the two groups. **B** GSVA based on the KEGG database. **C, D** Tumor mutation burden and TIDE score differences between the low- and high-risk groups. **E** Tumor-associated gene mutation burden in samples from the high-risk group. **F** Tumor-associated gene mutation burden in samples from the low-risk group. **G** Survival analysis of the H-TMB+high risk group, H-TMB+low risk group, L-TMB+high risk group, and L-TMB+low risk group.

Immune cell infiltration plays an important role in tumorigenesis and development [44, 45]. We investigated whether there were any differences in the genomes of patients in two risk cohorts. GO enrichment analysis revealed the enriched biological processes such as endothelial cell proliferation, lipid localization, and lipid transport. The cell component was primarily concentrated within the apical part of the cell, collagen-containing extracellular matrix, among others, while enriched molecular functions were secondary active transmembrane transporter and anion transmembrane transport functions (Fig. 8A). The two risk cohorts indicated significant variations in the immune cell infiltration-linked gene sets, such as immune checkpoint and T-cell co-inhibitors (Fig. 8B), suggesting a link across our risk model and immune cell infiltration. Since TMB and TIDE scores are very important in immunotherapy decision-making, TMB scores of downloaded and analyzed TCGA somatic mutation data of the patient cohort were determined; however, no notable variations were observed. Thus, the model based on m6A and cuproptosis had no correlation with TMB (Fig. 8C). Furthermore, TIDE scores were assessed to predict the efficiency of immune checkpoint blockade treatment, which revealed lower TIDE scores in the low-risk cohort. Thus, the high-risk cohort is more likely to exhibit better sensitivity to immunotherapy (p < 0.001), and our model can serve as an indicator for predicting TIDE (Fig. 8D). Moreover, median TMB scoring was used to group cases into the following cohorts: high risk and high TMB, high risk and low TMB, low risk and high TMB, and low risk and low TMB. TMB landscapes between the two subcohorts are shown in Fig. 8E, F. The survival assessment demonstrated a favorable prognosis in the low risk and low TMB cohort in comparison with other cohorts (Fig. 8G).

### NFE4 expression knockdown and its effect on ccRCC cells

We investigated the function of identified four m6A and cuproptosis-linked lncRNAs in ccRCC. Given that only LINC02154 and NFE4 expression was upregulated in ccRCC (Fig. 3I) and that LINC02154 has been described as a cuproptosis-linked gene and well-studied in various carcinomas including ccRCC [46–48], NFE4 was primarily focused.

**Figure. 9:**
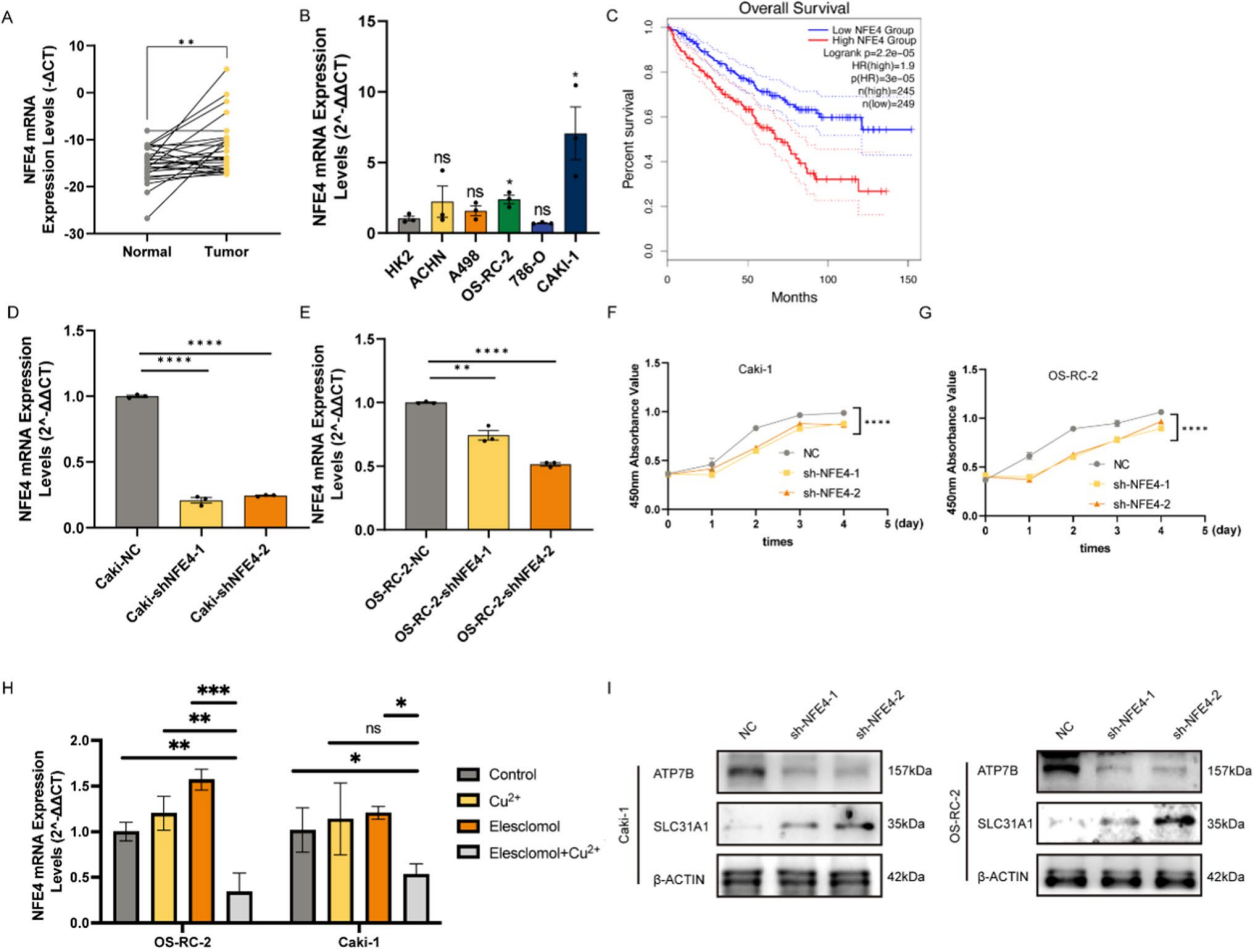
Functional experiments against NFE4 in Caki-1 and OS-RC-2 cells. **A** NFE4 was highly expressed in tumor tissues. **B** The expression of NFE4 in renal cancer cells was measured using real-time quantitative reverse transcription-PCR. **C** Survival analysis of the high NFE4 group and low NFE4 group. **D, E** The mRNA expression of NFE4 in Caki-1 and OS-RC-2 cells was measured using real-time quantitative reverse transcription-PCR. **F, G** Cell Counting Kit-8 assays were used to detect the effect of NFE4 knockdown on cell proliferation in Caki-1 and OS-RC-2 cells. **H** qRT-PCR shows the expression and regulation of NFE4 in Caki-1 and OS-RC-2 cells treated with drugs that induce cuproptosis for 24 h (n = 3). CuCl2 (2mM), Elesclomol (20 nM), both CuCl2 (2mM) and Elesclomol (20 nM). **I** Western blotting was used to determine the protein levels of ATP7B and SLC31A1 in Caki-1 and OS-RC-2 cells. One-way ANOVA, NC was used as a control group. Data are shown as the mean ± standard deviation from three independent experiments and were compared to the respective si-NC group. ****P < 0.0001; ***P < 0.001; **P < 0.01 and *P < 0.05.

The presence of any variation in NFE4 expression in the collected paired tissue and RCC cultures was assessed, which indicated that NFE4 was upregulated in ccRCC samples (Fig. 9A). NFE4 expression was also considerably upregulated in ccRCC cellular cultures as compared to control (HK2) cells (Fig. 9B). Moreover, high NFE4 expression patients had worse outcomes (Fig. 9C), and NFE4 was associated with T stage, grade, and patient vital status (Table 1).

**Table 1:** The characteristic of NFE4 in clear cell renal cell carcinoma

Characteristic	Level	Total (n = 514)	NFE4^*^		p Value
			High(257)	Low(257)	
Gender	Male	330	168	162	0.6455
	Female	184	89	95	
Age (median [IQR])		60.500 [52.000, 69.750]	60.000 [51.000, 69.000]	62.000 [52.000, 71.000]	0.1485
T	T1	257	120	137	0.0254^*^
	T2	66	28	38	
	T3	180	100	80	
	T4	11	9	2	
N	N0 + NX	498	249	249	1
	N1	16	8	8	
M	M0 + MX	435	213	222	0.3279
	M1	79	44	35	
Stage	I	251	117	134	0.1499
	II	54	22	32	
	III	123	68	55	
	IV	83	48	35	
	Not report	3	2	1	
Grade	1	13	6	7	0.0115^*^
	2	217	103	114	
	3	201	95	106	
	4	75	51	24	
	X	8	2	6	
Vital status	Alive	357	165	192	0.0043^*^
	Dead	154	92	62	
	Not report	3	0	3	

*The cutoff value for NFE4 is the median, specifically 0.1342. The data is from the TCGA-KIRC dataset

**Figure. 10:**
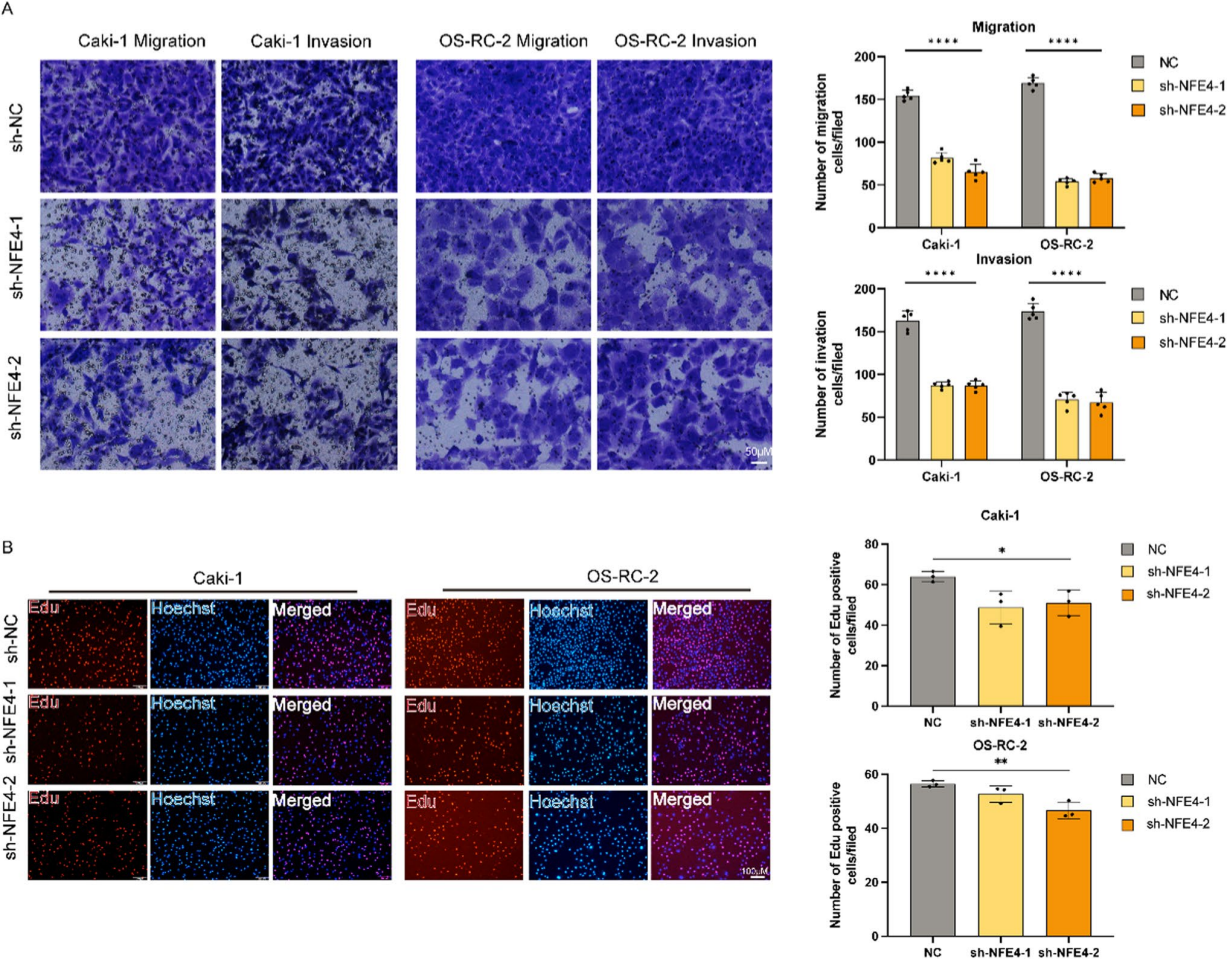
Functional experiments against NFE4 in Caki-1 and OS-RC-2 cells. **A** Representative images of migration and invasion assays for Caki-1 and OS-RC-2 cells. Transwell migration and invasion assays indicated that the migration and invasion abilities of NFE4 were weakened in the siRNA groups of Caki-1 and OS-RC-2 cells. **B** The effect of NFE4 on Caki-1 and OS-RC-2 cell proliferation was examined by EdU assay. One-way ANOVA, NC was used as a control group. Data are shown as the mean ± standard deviation from three independent experiments and were compared to the respective si-NC group. ****P < 0.0001; ***P< 0.001; **P< 0.01 and *P< 0.05.

To examine its effects on ccRCC, NFE4 was knocked down in RCC cell cultures via si-NFE4. qRT-PCR results confirmed the downregulated mRNA expression of NFE4 in Caki-1 (Fig. 9D) and OS-RC-2 cells (Fig. 9E) relative to the negative control. Then, a series of assessments were performed to determine if NFE4 influences carcinogenic properties. NFE4 expression knockdown inhibited the proliferation of both cell cultures (Fig. 9F and G). To demonstrate the close association of NFE4 with cuproptosis in renal cancer cells, qRT-PCR was performed to study its RNA expression and its regulatory role in the presence of copper ions and copper ionophore eleclomol in OS-RC-2 and Caki-1 cell lines. The results showed that the NFE4 gene was down-regulated in the presence of Cu^2+ ^or Elesclomol (Fig. 9H). Moreover, NFE4 was gradually elevated with the prolonged treatment of Elesclomol (Supplementary Figure S3), indicating its cuproptosis resistance role in KIRC. However, when treated with Cu^2+^ and Elesclomol simultaneously, the expression value of NFE4 was controversially downregulated. We speculated that this may be due to the tumor cell apoptosis induced by severe cuproptosis. Furthermore, Western blotting indicated that in the OS-RC-2 and Caki-1 cells, the copper transporter was altered after NFE4 knockout. The expression of the copper ingestion protein SLC31A1 increased, while the expression of the copper transport protein ATP7B decreased (Fig. 9I). Moreover, the migration and invasion ability of Caki-1 and OS-RC-2 cells were also reduced after NFE4 knockdown (Fig. 10A). Taken together, NFE4 is associated with the migration, proliferation, and invasive capabilities of ccRCC cells. The EdU assessment result further confirmed the significant decrease in the proliferation of these cells following NFE4 knockdown (Fig. 10B).

## Discussion

Scientists evaluated the pathophysiology of ccRCC, the most prevalent subtype of renal tumors, to identify potent treatment options and achieve favorable clinical outcomes. While surgical resection is a modest option and has offered favorable results in localized ccRCC, treatment outcomes of advanced/metastatic ccRCC have been unsatisfactory. Therefore, improvement in ccRCC patient’s outcomes warrants better patient prognosis and specific molecular profile.

The post-transcriptional modification m6A has a regulatory role in transcription, splicing, and translation. It influences the structures and functions of different lncRNAs [49]. Currently, 19 m6A regulators have been shown to modify specific lncRNAs in different cancers [50]. The literature confirms that m6A-modified lncRNAs affect tumorigenesis and cancer progression. Furthermore, lncRNAs act as competitive endogenous RNAs and affect aggressive tumor progression via m6A regulators. M6A modifications can modulate lncRNA functions by providing the m6A reader protein with a binding site. Moreover, it can regulate local RNA structures and enable specific RNA-binding proteins to recognize surrounding m6A residues. It has been observed that intra-cellular copper aggregation induces inhibition of aggregated lipid acylated proteins and Fe-S cluster proteins in the mitochondria, consequently causing proteinotoxic stress-induced death, a process termed copper toxicity [14]. Notably, intracellular copper accumulation relies upon transport facilitated by copper ionophores. Therefore, copper ionophores are crucial candidates for studying copper toxicity [28]. However, the roles of m6A- and cuproptosis-linked lncRNAs in ccRCC progression have not been fully elucidated and the knowledge of underlying biological mechanisms and prognostic biomarkers is still nascent [51, 52]. Given the pivotal functions of m6A, cuproptosis, and lncRNAs in ccRCC, this investigation developed a standalone risk model depending upon m6A- and cuproptosis-linked lncRNAs. Here, four prognostically relevant m6A- and cuproptosis-linked lncRNAs, including, NFE4, LINC02154, AL161782.1, and AL355835.1 were identified, which were employed for constructing a prognostic profile to pinpoint predictive accuracy for ccRCC case outcomes from TCGA. First, the patients were grouped into training and testing sets, subsequently, LASSO, multivariate Cox tests, and proportional hazard regression assessments were performed to identify prognoses-linked function for such ccRCC candidates. This investigation developed a prognosis risk score model, and its prediction potential was assessed by categorizing the patients into low-/high-risk subcohorts depending upon the median risk score. Consequently, a K–M survival test was performed, validating that the OS of the high-risk subcohort was less than the low-risk subcohort across all sets. This observation was consistent with the dataset outcomes of ROC curve evaluations. Furthermore, a prognostic nomogram was developed for quantitatively predicting the OS of ccRCC cases. In conclusion, such a prognostic model derived from m6A and cuproptosis-linked lncRNAs exhibited robust stability and stable prognostic predictive capabilities.

We determined the clinical value of these prognostic markers by integrating the risk scorings into clinicopathological characteristics and conducted uni-/multi-variate Cox regression and segmented assessments. Our findings revealed risk scoring, age, and grade function were independent prognosis parameters, suggesting m6A-lncRNAs and cuproptosis-lncRNAs prognostic markers could provide independent and reliable prediction of OS for patients with ccRCC. Moreover, according to the stratified assessment, the high-risk subcohort exhibited worse OS than the low-risk subcohort for multiple medical features. These results indicate the reliability and utility of prognostic indicators employed in our model.

We performed functional expression assessment for four m6A-associated lncRNAs, which showed that NFE4 and LINC02154 expression was upregulated in tumor tissues and they served as the risk factors in the high-risk cohort because of their overexpression in subcohort. Dataset outcomes for K-M survival assessment also demonstrated a correlation of upregulated NFE4 and LINC02154 with poorer survival outcomes. Thus, it can be speculated that these genes may act as tumor promoters in ccRCC. In contrast, AL161782.1 and AL355835.1 were downregulated in tumor tissue and served as protective factors, consistent with an increase in their expression within low-risk subcohort. Downregulation of AL161782.1 and AL355835.1 was associated with worse survival outcomes; therefore, they might act as tumor suppressors in ccRCC. This finding provides evidence for their possible association with ccRCC tumorigenesis and progression. Therefore, future studies should validate their functions and mechanism of action through in vitro experiments. Additionally, it was also uncovered that NFE4 promotes cell proliferation, migration, and invasion properties.

As a newly discovered cell death mode, cuproptosis and its related genes have gained unprecedented focus in tumor research. Numerous studies have highlighted the contributions of cuproptosis-related genes to uncontrolled tumor growth. Dihydrolipoamide Branched Chain Transacylase E2 (DBT), a key enzyme in transforming α-keto acid into acyl-CoA, served as a protective factor in cervical cancer [53]. FOXD2-AS1 and LINC02154 are the risk factors for KIRC patients [48, 54, 55]. Furthermore, some epigenetics regulators can modulate cuproptosis, including m6A. In gastric cancer, by increasing the FDX1 mRNA m6A modification, METTL16 could evaluate the cuproptosis sensitivity of cancer cells [56]. Zhu et.al. constructed a five-lncRNA risk model based on cuproptosis- and m6A-related lncRNAs [57]. In KIRC, LINC02154 is identified to be a cuproptosis-linked gene [47]. However, here, it was found that NFE4 was a novel cuproptosis- and m6A-related lncRNA. When treated with Cu^2+^ and elesclomol, the expression of NFE4 was significantly inhibited in KIRC cells, which was consistent with studies from Zhang et.al. [58].

However, our study still has some limitations. First, this investigation obtained the dataset to develop and validate the lncRNA prognostic signature only from TCGA. Second, this investigation only conducted preliminary studies on expression analyses of these four candidate lncRNAs, and no mechanistic studies were performed. Finally, the exact biological roles or specific pathways for candidate lncRNAs were not validated or identified, respectively. In future studies, we decided to validate our models in a real-world cohort, and the mechanism will be explored comprehensively.

In conclusion, this investigation employed the publicly available data of ccRCC patients to analyze functions for m6A and cuproptosis-linked lncRNAs in ccRCC pathogenesis. Furthermore, a prognostic risk profile was developed based on the four candidate lncRNAs, verifying model robustness and sensitivity. Additionally, a nomogram was constructed for the quantitative prediction of prognostic outcomes in ccRCC. Moreover, additional functional enrichment and mutation analyses were performed to support the acquired results. Overall, this investigation highlights NFE4 as a candidate lncRNA involved in ccRCC.

### Supplementary Information

Supplementary Material 1 (TXT 91 KB)

Supplementary Material 2 (ZIP 46 KB)

Supplementary Material 3 (PDF 224 KB)

Supplementary Material 4 (PDF 256 KB)

Supplementary Material 5 (PDF 205 KB)

Supplementary Material 6 (DOCX 20 KB)

Supplementary Material 7 (TXT 42 KB)

Supplementary Material 8 (ZIP 1712 KB)

## Data Availability

The data used to support the findings of this study are available from the corresponding author upon request.
