# Supplementary material for: Bioinformatics analysis and experimental validation of m6A and cuproptosis-related lncRNA NFE4 in clear cell renal cell carcinoma
Source: Discov Oncol. 2024 May 26;15:187. doi: 10.1007/s12672-024-01023-y (PMC11128431; doi:10.1007/s12672-024-01023-y)
Supplement: Supplementary file 3 — Supplementary Material 3 (PDF 224 KB) [file 12672_2024_1023_MOESM3_ESM.pdf]

A

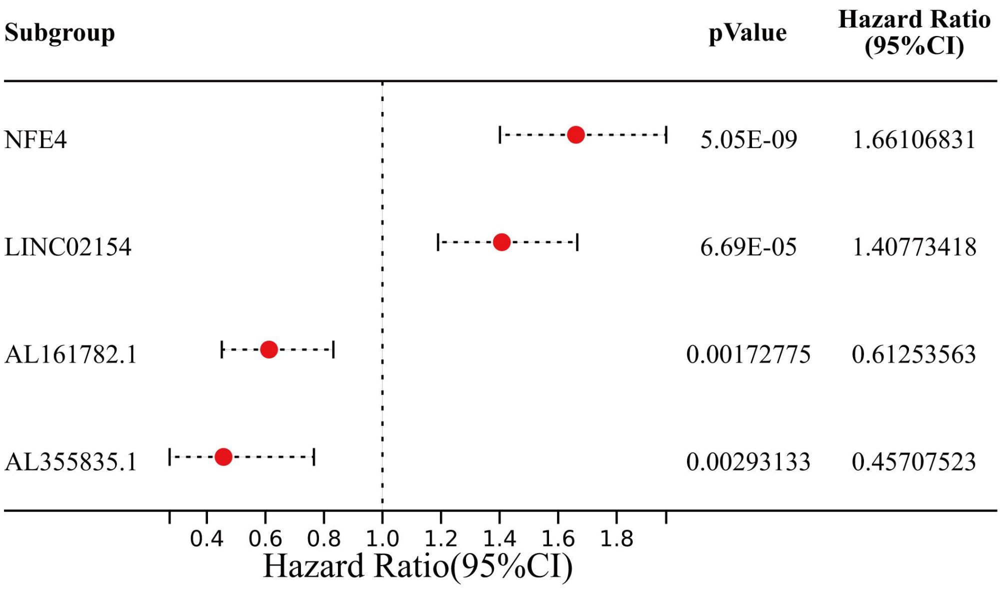

Figure S1. HR Forest Diagram.（A）Prognosis-related multivariate cox regression analysis of lncRNAs.
