# Supplementary material for: Bioinformatics analysis and experimental validation of m6A and cuproptosis-related lncRNA NFE4 in clear cell renal cell carcinoma
Source: Discov Oncol. 2024 May 26;15:187. doi: 10.1007/s12672-024-01023-y (PMC11128431; doi:10.1007/s12672-024-01023-y)
Supplement: Supplementary file 4 — Supplementary Material 4 (PDF 256 KB) [file 12672_2024_1023_MOESM4_ESM.pdf]

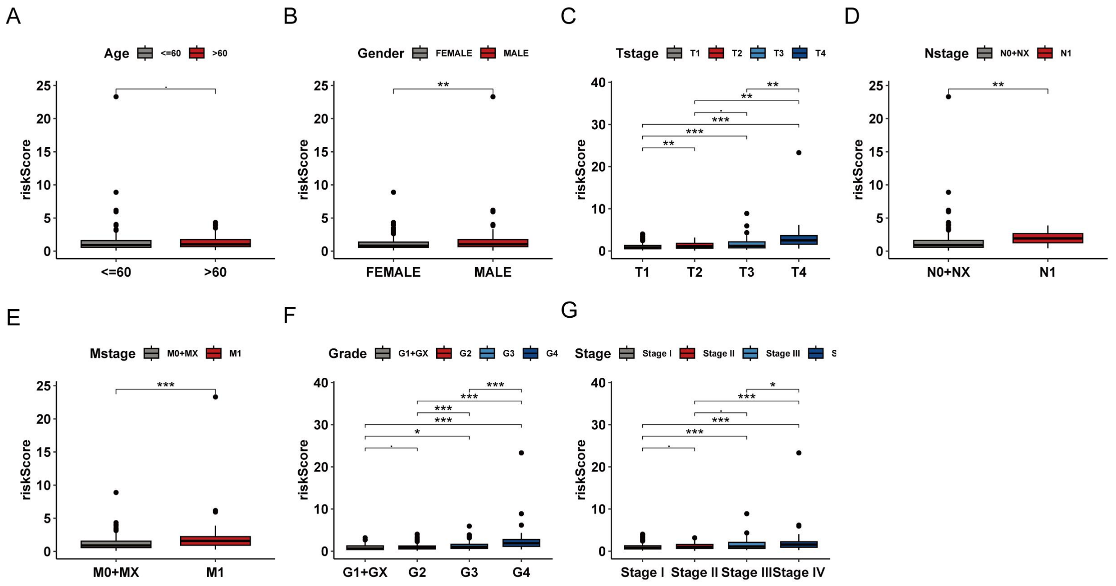

Figure S2. Wilcoxon test.（A-G）The differences in risk scores between subgroups were compared with clinical characteristics such as age, gender, tumor differentiation, and TNM stage.
