# Supplementary figures and images for: Bioinformatics analysis and experimental validation of m6A and cuproptosis-related lncRNA NFE4 in clear cell renal cell carcinoma
Source: Discov Oncol. 2024 May 26;15:187. doi: 10.1007/s12672-024-01023-y (PMC11128431; doi:10.1007/s12672-024-01023-y)

A

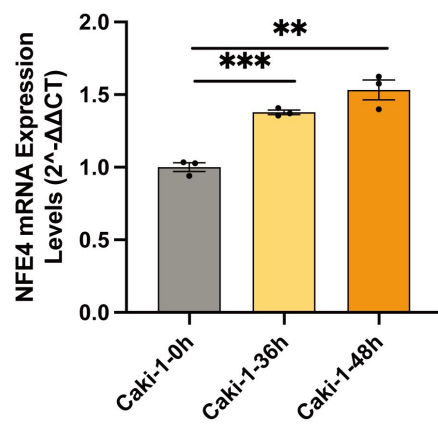

B

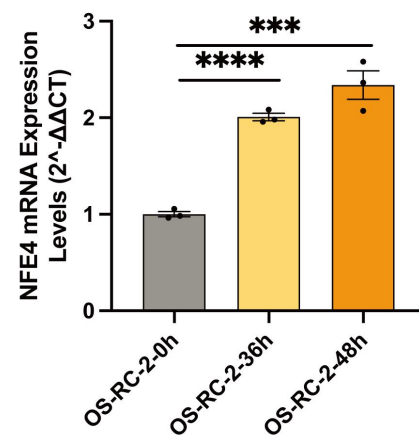

Figure S3. (A-B) Expression of NFE4 RNA at different treatment times of 20 nM Elesclomol.

Supplement: Supplementary file 5 — Supplementary Material 5 (PDF 205 KB) [file 12672_2024_1023_MOESM5_ESM.pdf]

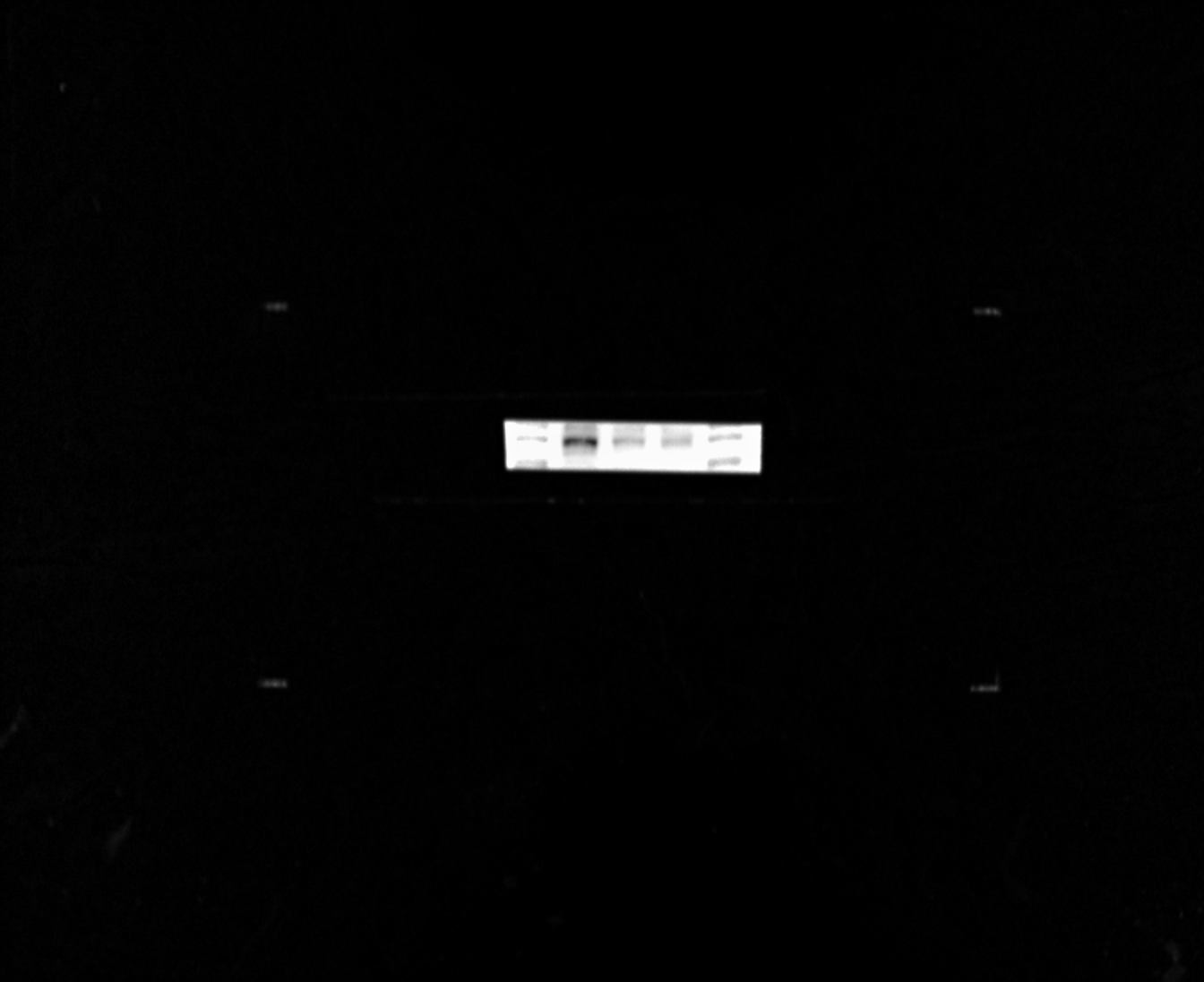

Supplement: Supplementary file 8 — Supplementary Material 8 (ZIP 1712 KB) [file 12672_2024_1023_MOESM8_ESM.zip › Western blots/Caki-1/ATP7B.tif]

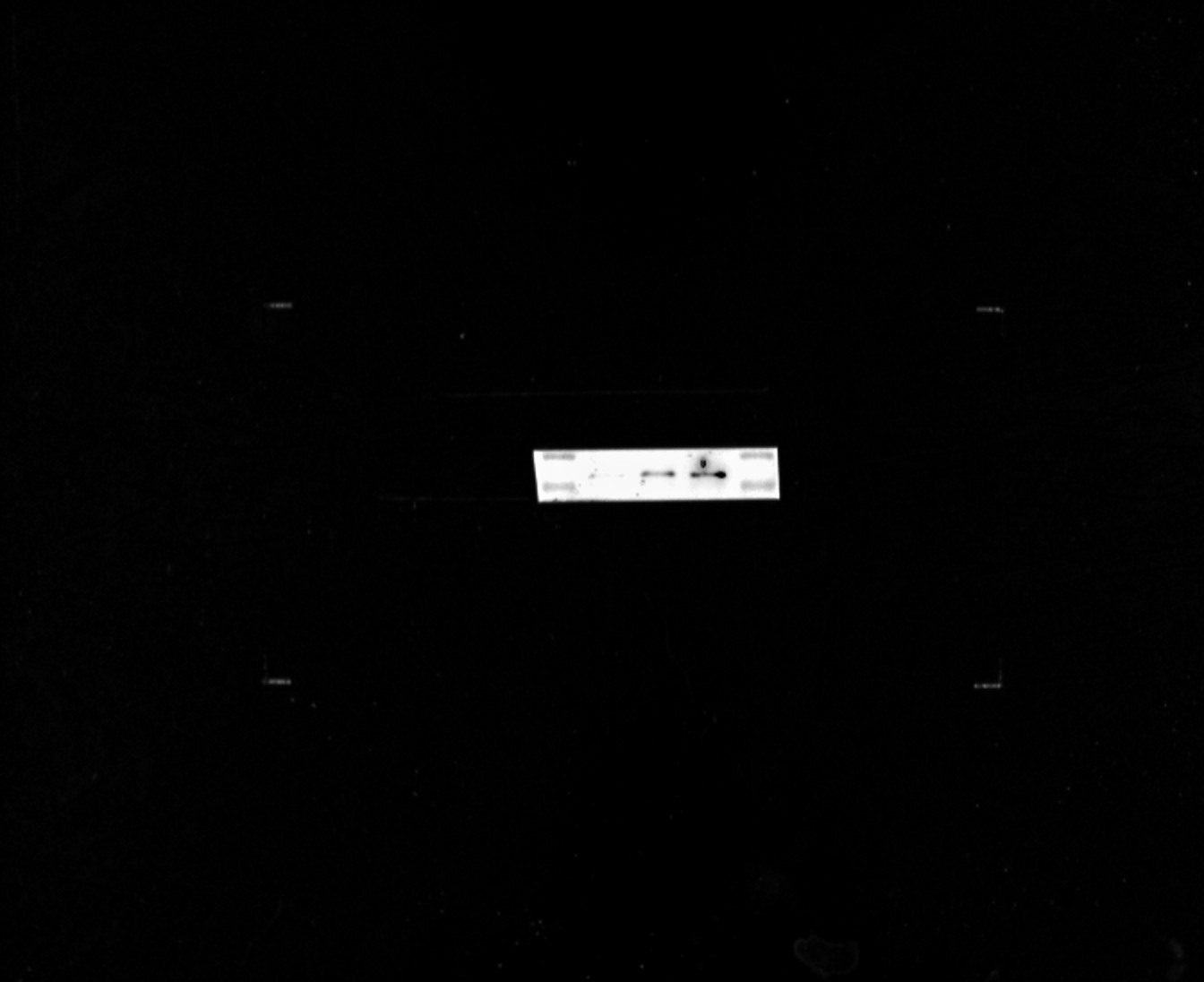

Supplement: Supplementary file 8 — Supplementary Material 8 (ZIP 1712 KB) [file 12672_2024_1023_MOESM8_ESM.zip › Western blots/Caki-1/SCL31A1.tif]

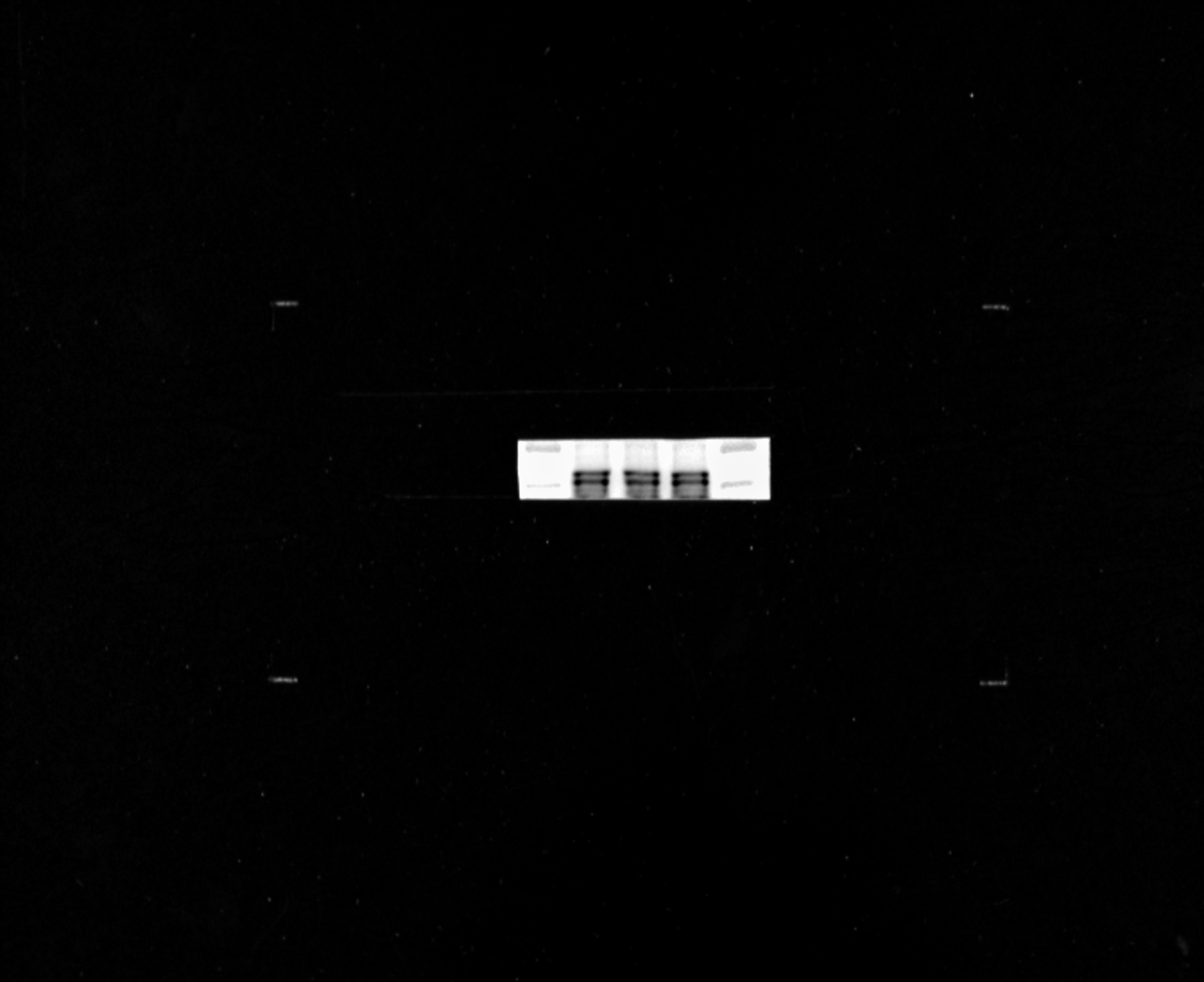

Supplement: Supplementary file 8 — Supplementary Material 8 (ZIP 1712 KB) [file 12672_2024_1023_MOESM8_ESM.zip › Western blots/Caki-1/a┬-ACTIN.tif]

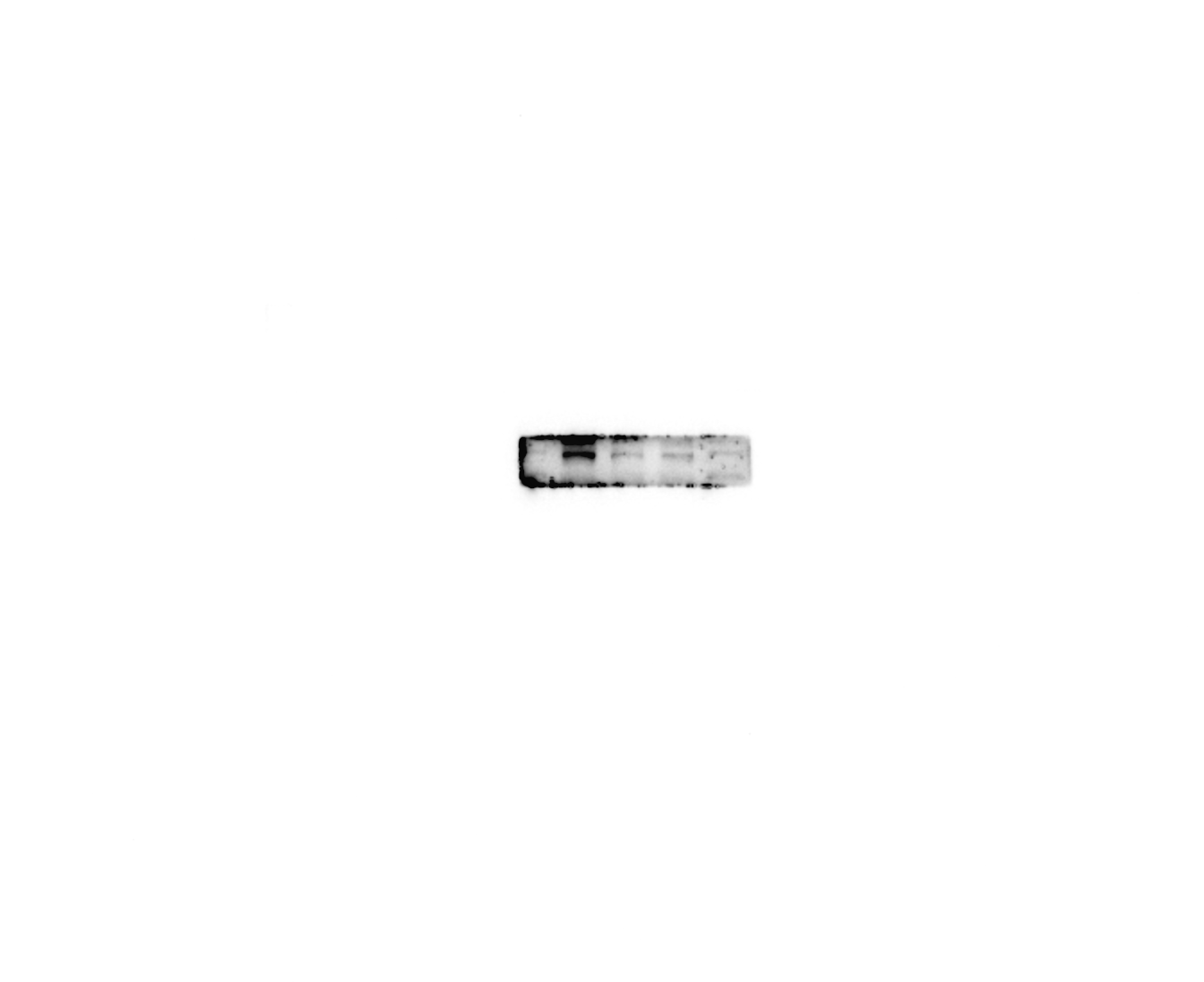

Supplement: Supplementary file 8 — Supplementary Material 8 (ZIP 1712 KB) [file 12672_2024_1023_MOESM8_ESM.zip › Western blots/OS-RC-2/ATP7B.tif]

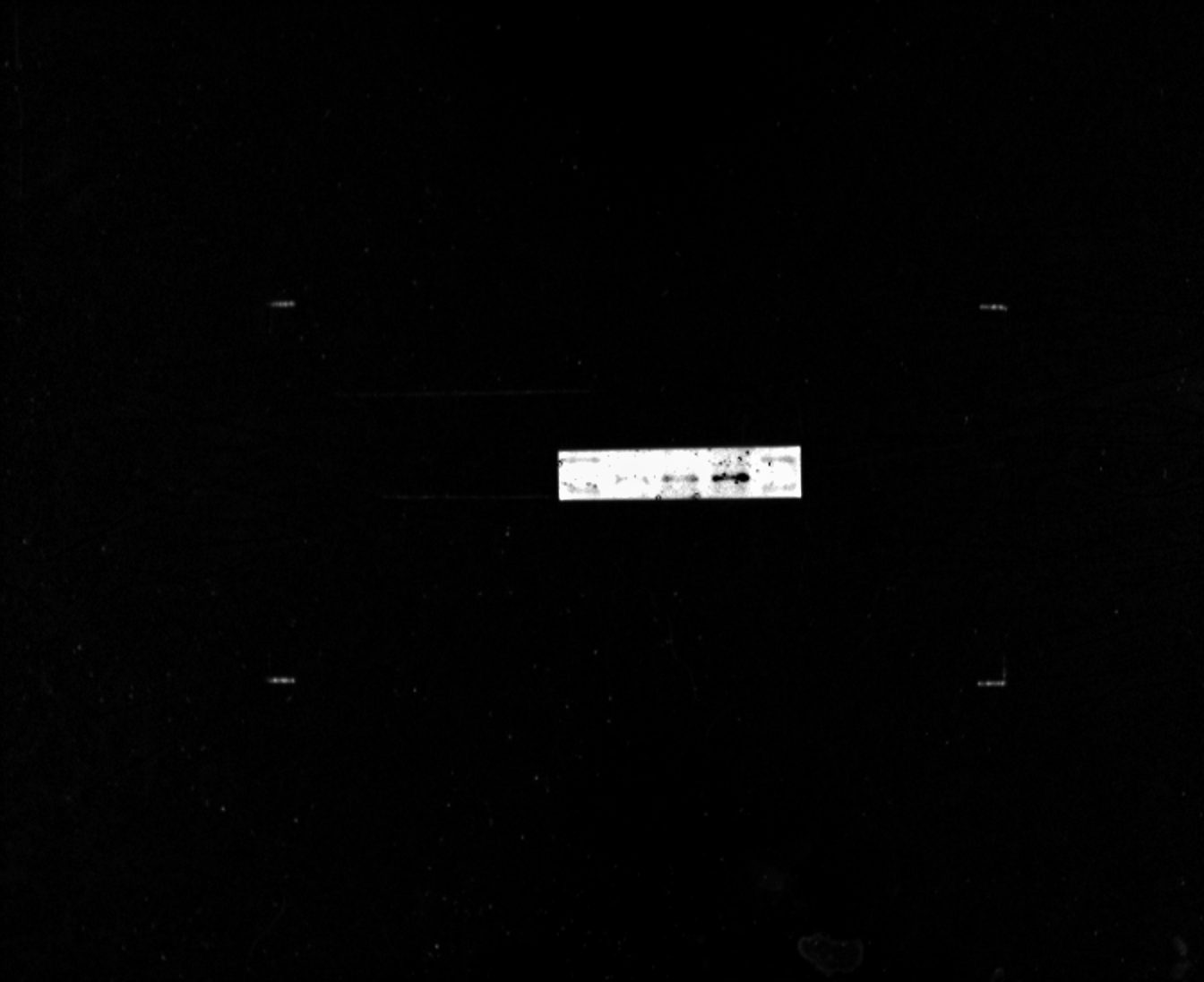

Supplement: Supplementary file 8 — Supplementary Material 8 (ZIP 1712 KB) [file 12672_2024_1023_MOESM8_ESM.zip › Western blots/OS-RC-2/SLC31A1.tif]

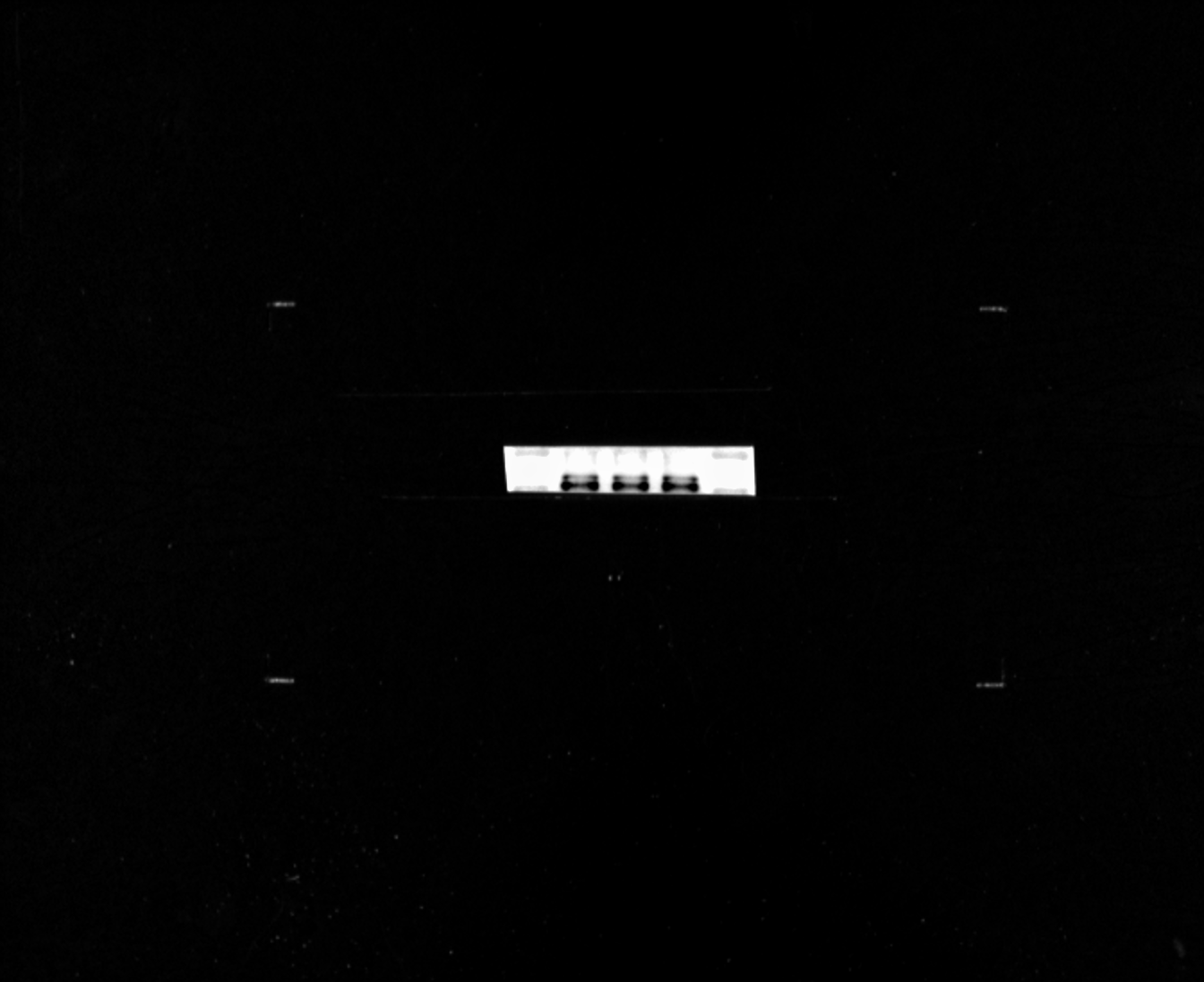

Supplement: Supplementary file 8 — Supplementary Material 8 (ZIP 1712 KB) [file 12672_2024_1023_MOESM8_ESM.zip › Western blots/OS-RC-2/a┬-ACTIN.tif]
