# Supplementary material for: Bioinformatics analysis and experimental validation of m6A and cuproptosis-related lncRNA NFE4 in clear cell renal cell carcinoma
Source: Discov Oncol. 2024 May 26;15:187. doi: 10.1007/s12672-024-01023-y (PMC11128431; doi:10.1007/s12672-024-01023-y)
Supplement: Supplementary file 6 — Supplementary Material 6 (DOCX 20 KB) [file 12672_2024_1023_MOESM6_ESM.docx]

Table S1 Clinical characteristics among the two cohorts.

| Covariates | **Type** | **Total (n=370)** | **Test (n=185)** | **Train (n=185)** | **Pvalue** |
| --- | --- | --- | --- | --- | --- |
| Age | <=60 | 177(47.84%) | 93(50.27%) | 84(45.41%) | 0.4051 |
|  | >60 | 193(52.16%) | 92(49.73%) | 101(54.59%) |  |
| Gender | FEMALE | 121(32.7%) | 67(36.22%) | 54(29.19%) | 0.1836 |
|  | MALE | 249(67.3%) | 118(63.78%) | 131(70.81%) |  |
| Grade | G1 | 55(14.86%) | 25(13.51%) | 30(16.22%) | 0.8011 |
|  | G2 | 177(47.84%) | 91(49.19%) | 86(46.49%) |  |
|  | G3 | 121(32.7%) | 59(31.89%) | 62(33.51%) |  |
|  | G4 | 12(3.24%) | 7(3.78%) | 5(2.7%) |  |
|  | unknow | 5(1.35%) | 3(1.62%) | 2(1.08%) |  |
| Stage | Stage I | 171(46.22%) | 83(44.86%) | 88(47.57%) | 0.4545 |
|  | Stage II | 85(22.97%) | 46(24.86%) | 39(21.08%) |  |
|  | Stage III | 85(22.97%) | 41(22.16%) | 44(23.78%) |  |
|  | Stage IV | 5(1.35%) | 4(2.16%) | 1(0.54%) |  |
|  | unknow | 24(6.49%) | 11(5.95%) | 13(7.03%) |  |
| T | T1 | 181(48.92%) | 88(47.57%) | 93(50.27%) | 0.5361 |
|  | T2 | 93(25.14%) | 52(28.11%) | 41(22.16%) |  |
|  | T3 | 80(21.62%) | 39(21.08%) | 41(22.16%) |  |
|  | T4 | 13(3.51%) | 5(2.7%) | 8(4.32%) |  |
|  | unknow | 3(0.81%) | 1(0.54%) | 2(1.08%) |  |
| N | N0 | 252(68.11%) | 128(69.19%) | 124(67.03%) | 0.1472 |
|  | N1 | 4(1.08%) | 4(2.16%) | 0(0%) |  |
|  | unknow | 114(30.81%) | 53(28.65%) | 61(32.97%) |  |
| M | M0 | 266(71.89%) | 138(74.59%) | 128(69.19%) | 0.6784 |
|  | M1 | 4(1.08%) | 3(1.62%) | 1(0.54%) |  |
|  | unknow | 100(27.03%) | 44(23.78%) | 56(30.27%) |  |
